# Identification and expression analysis of *WRKY* gene family under drought stress in peanut (*Arachis hypogaea* L.)

**DOI:** 10.1371/journal.pone.0231396

**Published:** 2020-04-09

**Authors:** Nannan Zhao, Meijing He, Li Li, Shunli Cui, Mingyu Hou, Liang Wang, Guojun Mu, Lifeng Liu, Xinlei Yang

**Affiliations:** College of Agronomy, Hebei Agricultural University, Baoding, Hebei, China; ICAR - National Research Center on Plant Biotechnology, INDIA

## Abstract

WRKY transcription factors play crucial roles in regulation mechanism leading to the adaption of plants to the complex environment. In this study, AhWRKY family was comprehensively analyzed using bioinformatic approaches in combination with transcriptome sequencing data of the drought-tolerant peanut variety ‘L422’. A total of 158 *AhWRKY* genes were identified and named according to their distribution on the chromosomes. Based on the structural features and phylogenetic analysis of AhWRKY proteins, the *AhWRKY* family members were classified into three (3) groups, of which group II included five (5) subgroups. Results of structure and conserved motifs analysis for the *AhWRKY* genes confirmed the accuracy of the clustering analysis. In addition, 12 tandem and 136 segmental duplication genes were identified. The results indicated that segmental duplication events were the main driving force in the evolution of *AhWRKY* family. Collinearity analysis found that 32 gene pairs existed between *Arachis hypogaea* and two diploid wild ancestors (*Arachis duranensis* and *Arachis ipaensis*), which provided valuable clues for phylogenetic characteristics of *AhWRKY* family. Furthermore, 19 stress-related *cis*-acting elements were found in the promoter regions. During the study of gene expression level of *AhWRKY* family members in response to drought stress, 73 differentially expressed *AhWRKY* genes were obtained to have been influenced by drought stress. These results provide fundamental insights for further study of *WRKY* genes in peanut drought resistance.

## Introduction

The WRKY transcription factors are one of the largest families of transcriptional regulators in plants and form an integral part of signaling networks that regulate many plant processes [1]. They are important to plant growth by regulating development and responding to stresses [1–7]. WRKY proteins contain at least one WRKY domain which consists of approximately 60 amino acids with a highly conserved WRKYGQK heptapeptide at the N-terminus, and include a C_2_H_2_ (C–X_4–5_–C–X_22–23_–H–X–H) or C_2_HC (C–X_7_–C–X_23_–H–X–C) zinc-finger motif in C-terminus. *WRKY* transcription factors regulate gene expression by exclusively binding to W-box, which is a *cis*-element in the promoter regions of target genes [2]. Based on the number and characteristics of WRKY domains and zinc finger structure, respectively, the WRKY proteins can be divided into three (3) main groups, namely group I, II and III. The group I contains two (2) WRKY domains and a zinc finger motif of C_2_H_2_ type (C-X_4_-C-X_22–23_-H-X_1_-H), group II has a single WRKY domain and a zinc finger motif of C_2_H_2_ type (C-X_4–5_-C-X_23_-H-X_1_-H) and group III consists of a single WRKY domain and a zinc-binding motif of C_2_HC type (C-X_7_-C-X_23_-H-X_1_-C). Group II is further partitioned into five (5) subgroups (IIa, IIb, IIc, IId, and IIe) based on the additional conserved structural motifs of the WRKY structure [8].

A large number of studies on a functional analysis of *WRKY* genes have indicated the involvement of WRKY transcription factors in plant defense regulatory networks and developmental processes. SPF1 was the first WRKY transcription factor to be reported in sweet potato [9]. And with the continuous development of genome sequencing and other high throughput technologies, members of the *WRKY* gene family are being identified and perform diverse roles in various species including *Ipomoea batatas* [9], *Arabidopsis thaliana* [10], *Oryza sativa* [11], *Glycine max* [12], *Gossypium raimondii* [13], *Lotus japonicas* [14], *Triticum aestivum* [15], *Vitis vinifera* [16], *Ananas comosus* [17], *Arachis duranensis* and *Arachis ipaensis* [18]. For instance, in *Cucumis*, the expression of *CsWRKY50* could positively regulate resistance to *Pseudoperonospora cubensis* involving multiple signaling pathways [19]. In rice, it has been reported that *OsWRKY71* was up-regulated by defense signaling molecules, such as salicylic acid (SA), methyl jasmonate (MeJA) and pathogen infection, indicating that this member of the *WRKY* gene family might be involved in biotic stress response [20]. *OsWRKY53* has also been found to positively regulate the brassinosteroid (BR) signaling and mediate the cross-talk between the hormone and other signaling pathways [21]. And *OsWRKY78* member played an important role in regulation of stem elongation and seed development [22]. In *Arabidopsis*, *AtWRKY8* may participate in defensive response to the infection of crucifer-infecting tobacco mosaic virus (TMV-cg) as the virus inhibited its expression [23]. It has also been reported that *AtWRKY75* is involved in the regulation of Phosphate (Pi) deficiency response and root development [24]. *AtWRKY6* has also been found to be related to the triggering of senescence process and defense response [25]. In transgenic *Arabidopsis thaliana*, overexpression of *AtWRKY13* resulted in decreased Cadmium (Cd) accumulation with increased tolerance to it [26]. In grape, the expression of *VvWRKY30* could improve salt tolerance by regulating the elimination of reactive oxygen and the accumulation of osmoticum [27]. Overexpression of *TaWRKY2* conferred strong drought tolerance to transgenic wheat [28]. And *ScWRKY1* was highly and transiently expressed in fertilized ovules bearing late torpedo-staged embryos of potato, revealing a key role during embryogenesis [29].

Despite peanut (*Arachis hypogaea* L.) being one of the most important oil crops in the world and widely cultivated in the tropical and subtropical regions, its yields, particularly on farmers’ fields, are still low which is largely due to biotic and abiotic stresses. Drought is one of the major abiotic stress factors in limiting peanut production. It affects the physiology, biochemistry and molecular biology of plants [30–32]. And with peanut mainly cultivated under rainfed conditions by resource-poor farmers, breeding for drought-resistant peanut cultivars is the most economical and effective way to reduce the yield loss caused by menace. However, drought resistance in itself is a complex trait governed by a large number of genes [33, 34]. As a result, the mining and identification of potential genes associated with this trait will be of great importance for the development and subsequent cultivation of drought-resistant peanut varieties. Also, in view of the important roles played by WRKY transcription factors in stress response, a functional analysis of these transcription factors could offer an alternative means to improve peanut stress tolerance in general.

Information about the *WRKY* gene family has been characterized in the two wild ancestral species of peanut. In a previous study, 75 AdWRKYs from *A*. *duranensis* and 77 AiWRKYs from *A*. *ipaensis* were identified through bioinformatic approaches and *Arachis WRKY* proteins potentially controlling disease-resistance were deduced [18]. So far, the whole genome sequence of cultivated peanut (*Arachis hypogaea* cv. Tifrunner) has been completely sequenced [35] and released (http://www.peanutbase.org/). However, *WRKY* gene family involved in drought-responsive processes has not been systematically identified in cultivated peanut. In this study, the sequence features, chromosomal distribution, phylogenetic relationships, *cis*-elements, gene duplications, and synteny of *AhWRKYs* were comprehensively analyzed through bioinformatic methods. The gene expression level of *WRKY* gene family members in response to drought stress on the basis of the transcriptome sequencing data was also studied. The results will provide valuable information for further functional investigations of the *AhWRKY* gene family and preliminary knowledge of *AhWRKYs* potentially involved in drought resistance.

## Materials and methods

### Identification of WRKYs

Two approaches were used in the process of identifying *WRKYs*. In the first approach, the genome of *Arachis hypogaea* cv. Tifrunner was downloaded from the peanut genome database (PeanutBase) (https://www.peanutbase.org). Also, a Pfam file with multiple sequence alignments and a hidden Markov model (HMM) corresponding to the WRKY domain (PF03106) was downloaded from the Pfam database (http://pfam.xfam.org/). HMMER 3.0 was used to search for *WRKY* genes from the downloaded genome based on the default parameters at a probability value of 0.01. In the second approach, WRKY protein sequences for *Arabidopsis* (AtWRKY) were downloaded from the *Arabidopsis* Information Resource website (http://www.arabidopsis.org/), whiles WRKY sequences of *A*. *duranensis* (AdWRKY) and *A*. *ipaensis* (AiWRKY) were obtained from previous study [18]. The WRKYs from the second approach were used to validate those obtained from the first approach. After then, all validated candidate genes with probable WRKY domain were further validated using PFAM and SMART programs (http://smart.embl-heidelberg.de) to retain the only sequences with WRKY domain.

Additionally, the primary structure parameters of the genes (length of sequences, molecular weight, and isoelectric point) were analyzed using the ExPASy website (http://web.expasy.org/protparam/). The prediction of subcellular location of identified *WRKY* proteins was performed using Plant-mPLoc [36], ProtComp 9.0 and WoLF PSORT (http://www.csbio.sjtu.edu.cn/bioinf/plant-multi; http://linux1.softberry.com/berry.phtml?topic=protcomppl&group=programs&subgroup=proloc; https://wolfpsort.hgc.jp).

### Sequence alignment and phylogenetic analysis

Multiple sequence alignment of *Arachis hypogaea* WRKYs (AhWRKYs) domain sequences was executed using the Clustal X 2.1 program with default parameters [37] and results were colored using GeneDoc. Newly identified AhWRKYs were clustered into different groups based on the classification schemes of AtWRKYs, AdWRKYs, and AiWRKYs [8, 18]. The phylogenetic tree was generated and viewed using MEGA 7.0 with Maximum Likelihood (ML) method and 1000 replicates [38]. Gene structures were generated *via* an online tool GSDS (http://gsds.cbi.pku.edu.cn/) by comparing CDS of *AhWRKYs* with their corresponding full-length sequences [39]. The 1500 bp upstream of the coding region of *AhWRKYs* were selected and submitted to PlantCARE (http://bioinformatics.psb.ugent.be/webtools/plantcare/html) for predicting the *cis*-regulatory element of promoter [40]. The conserved motifs in the AhWRKYs were identified statistically using the MEME program (http://alternate.meme-suite.org/tools/meme) with default settings except the maximum number of motifs to be found which was set at 10 [41].

### Chromosomal distribution and gene duplication of *AhWRKYs*

Positional information of all candidate *AhWRKYs* was retrieved from the PeanutBase, and the locations were drafted using MG2C (http://mg2c.iask.in/mg2c_v2.0). Duplication events of genes were analyzed by means of Multiple Collinearity Scan toolkit (MCScanX) with the default parameters [42]. In order to exhibit the orthologous *WRKY* genes between peanut and other selected species, syntenic analysis maps were constructed using Circos software [43].

### Plant materials used and drought treatment

Two peanut cultivars, L422 (drought-tolerant) and L677 (drought-sensitive) were used for this study. Seeds were planted in rainout shelters under well-watered conditions in Baoding, China. The relative moisture content of the soil was maintained at 70–75% until the plants reached the reproductive phase (pegging stage) which coincided with 60 days after planting (DAP). At pegging, each plot of the two cultivars was split into two units to set up the control and treatment groups. The control group continued to be under well-watered conditions while the treatment group received no irrigation. Fully expanded leaves from the main stem (third nodal) were randomly selected after 20 days (80 DAP) of exposure. Fresh leaves of control and treatment plants were collected at 80, 85, 95, and 100 DAP, respectively. Three (3) independent samples were collected and used as biological replicates. The samples were immediately frozen in liquid nitrogen and stored at -80°C for subsequent analyses.

### Physiological index measurements

Physiological parameters were measured on the L422 and L677 plants both under well-watered and drought-stress conditions. The level of lipid peroxidation (MDA content) in the leaves was determined by Thiobarbituric acid (TBA) method [44]. Additionally, leaf superoxide dismutase (SOD) activity was measured spectrophotometrically at 560 nm according to a previously reported method [45]. Soluble protein content of samples was also measured by Coomassie Brilliant Blue method [46]. Each parameter measurement on each cultivar under each treatment group was replicated three (3) times using independent but parallel approaches. Student’s *t*-test was performed to determine the significance of treatment differences using Origin 8.0 software version v6.1052 (B232) (OriginLab Corp, Northhampton, MA, USA). Differences were considered significant if *p*-value was less than 0.05.

### Total RNA extraction, cDNA library construction, and RNA-Seq

The isolation of total RNA from non-stressed and stressed plant leaves was optimized according to the instruction manual of the Trizol Reagent (Invitrogen, Carlsbad, CA, USA). RNA degradation and contamination were monitored on 1% agarose gel and its quality was evaluated using NanoDrop ND-1000 spectrophotometer (NanoDrop Technologies Inc., Wilmington, DE, USA). The cDNA library construction and RNA sequencing were carried out on the Illumina HiseqTM 2500 platform using the 125-bp paired-end sequencing protocol of Illumina by Biomarker Technology Co. (Beijing, China).

### Sequencing reads processing and mapping

Raw reads in a FASTA format were first processed using in-house Perl scripts. In this step, clean reads (clean data) was obtained by removing reads with low quality and containing adapters, and ploy-N. Q20 (99% base call accuracy), Q30 (99.9% base call accuracy), GC-content and sequence duplication level of the clean data were calculated. All the downstream analyses were based on high-quality clean data. Qualified clean reads were then mapped to the peanut reference genome sequence (Tifrunner.gnm1.ann1.CCJH) using a spliced aligner Tophat 2.0 software [47]. Only reads with a perfect match or only one mismatch were further analyzed and annotated according to the reference genome. Quantification of gene expression was calculated as FPKM (fragments per kilobase of exon model per million mapped reads) and the FPKM values were log_2_ transformed [48]. The aligned reads were analyzed using StringTie [49], and after then the assembled transcripts were further filtered using the FPKM value > 1.

### Gene expression analysis and functional annotation of *AhWRKYs*

To identify differential expression genes (DEGs) between two different samples, the expression level of each transcript was calculated according to the FPKM method. EBSeq R software package (4.0) was used to execute gene differential expression analysis of two or more biological conditions in an RNA-seq experiment based on the negative binomial distribution model [50]. In case of no biological replicates, the package would estimate the variances of RNA-seq data by pooling similar genes together. *p*-values obtained *via* EBseq were further corrected using the Benjamini-Hochberg procedure [51], and the corrected *p*-values were used to determine the false discovery rate (FDR). Genes were considered to be differentially expressed when the value of log_2_ Fold Change was >1 or <-1 with an FDR value less than 0.05. To analyze the potential function of genes, all the genes were functionally annotated using Gene Ontology (GO) (http://www.geneontology.org/) and Kyoto Encyclopedia of Genes and Genomes (KEGG) (http://www.kegg.jp/) databases. GO functional classification enrichment analysis was performed using the OmicShare tools, an online platform for data analysis (http://www.omicshare.com/tools). Firstly, all the differentially expressed *WRKY* genes were mapped to GO terms in the Gene Ontology database, and gene numbers were calculated for every term. And then the differentially expressed *WRKYs* with significantly enriched GO terms were compared to the genome background and defined by hypergeometric test. Similarly, for each KEGG pathway, the numbers of differentially expressed *WRKYs* were compared to the entire reference gene set by hypergeometric tests using the OmicShare tools to determine the pathways enriched for differentially regulated genes. The calculated *p*-value was corrected through FDR Correction, taking FDR ≤ 0.05 as a threshold. The heatmap of *AhWRKYs* expression was also created using the OmicShare tools. All transcriptome data have been deposited at the National Center for Biotechnology Information (https://www.ncbi.nlm.nih.gov/sra/PRJNA544421).

### Validation of gene expression

Eleven (11) *AhWRKY* genes obtained by Illumina RNA-seq that showed differentially expressed and contained *cis*-recognition elements involved in the abiotic/biotic stress were randomly selected for validation by quantitative real-time PCR (qRT-PCR). All gene-specific primers were designed by Wcgene Biotech (Shanghai, China) (S1 Table). The housekeeping gene *EF1b* [52] was used as an internal control gene for qRT-PCR normalization. The qRT-PCR was run on the LightCycler® 96 instrument using Fast Super EvaGreen® qPCR Master Mix (US Everbright®Inc., China) based on the manufacturer’s instructions. The amplification program was set as follows: 95°C for 2 min followed by 45 cycles of 95°C for 5 s and 60°C for 1 min. Three (3) and two (2) biological and technical repetitions, respectively, were used for each gene sample. All data from qRT-PCR amplification were calculated with 2^−△△CT^ method [53]. All expression data were analyzed using the Origin 8.0 software version v6.1052 (B232) (OriginLab Corp, Northhampton, MA, USA).

## Results

### Identification of WRKY proteins in peanut cultivars used

A total of 158 members of WRKY family were identified using bioinformatic approaches, mapped onto chromosomes and were designated from *AhWRKY1* to *AhWRKY158* based on their locations on the respective chromosome (S2 Table). The particularizations including open reading frame lengths (ORF), amino acid numbers, molecular weights (MW), isoelectric point (pI), and the predicted subcellular locations are all shown in S2 Table. The lengths of the 158 identified AhWRKY proteins varied from 128 (AhWRKY29) to 1345 (AhWRKY54) amino acids. Additionally, the pI and MW ranged from 4.92 (AhWRKY43) to 9.92 (AhWRKY85) and from 14.9 kDa to 148.6 kDa, respectively. Results from the predicted subcellular localization showed 155 AhWRKY proteins were located in the nuclear region, whereas one protein was located in the cell membrane. In addition, two (2) proteins were located in both the cell membrane and the nuclear region, which suggests they might have special biological functions. The detailed information, including gene loci number, conserved motif, zinc finger domain pattern and gene grouping are also given in S2 Table.

### Multiple sequence alignment, phylogenetic tree analysis and group identification

To investigate the evolutionary relationship and classification of *AhWRKY*s, a total of 158 WRKY domains spanning approximately 60 amino acids were executed. For different groups, seven (7) *Arabidopsis* proteins were selected randomly as representative sequences (AtWRKY33; 60; 47; 48; 11; 22; 55). Detailed structures about the WRKY domain and zinc finger type are displayed in S1 Fig. One hundred and forty-two (142) of the 158 AhWRKY proteins contained WRKYGQK sequence which is a highly conserved sequence. However, six (6) variants of this conserved WRKY motif including GRKYGQK, WRKYGEK, WRKYGKK, WRKYGRK, WRKYDKK and WHKYGKK were distributed in subgroup IIc and I, respectively. Strikingly, C2HY, the zinc finger motif form variation was identified in *AhWRKY13*. Also, the *WRKY* sequences that had lost the *WRKYGQK* motif or the zinc finger motif were distributed in subgroups IIa, IIb, IIc and I, respectively.

**Fig 1:**
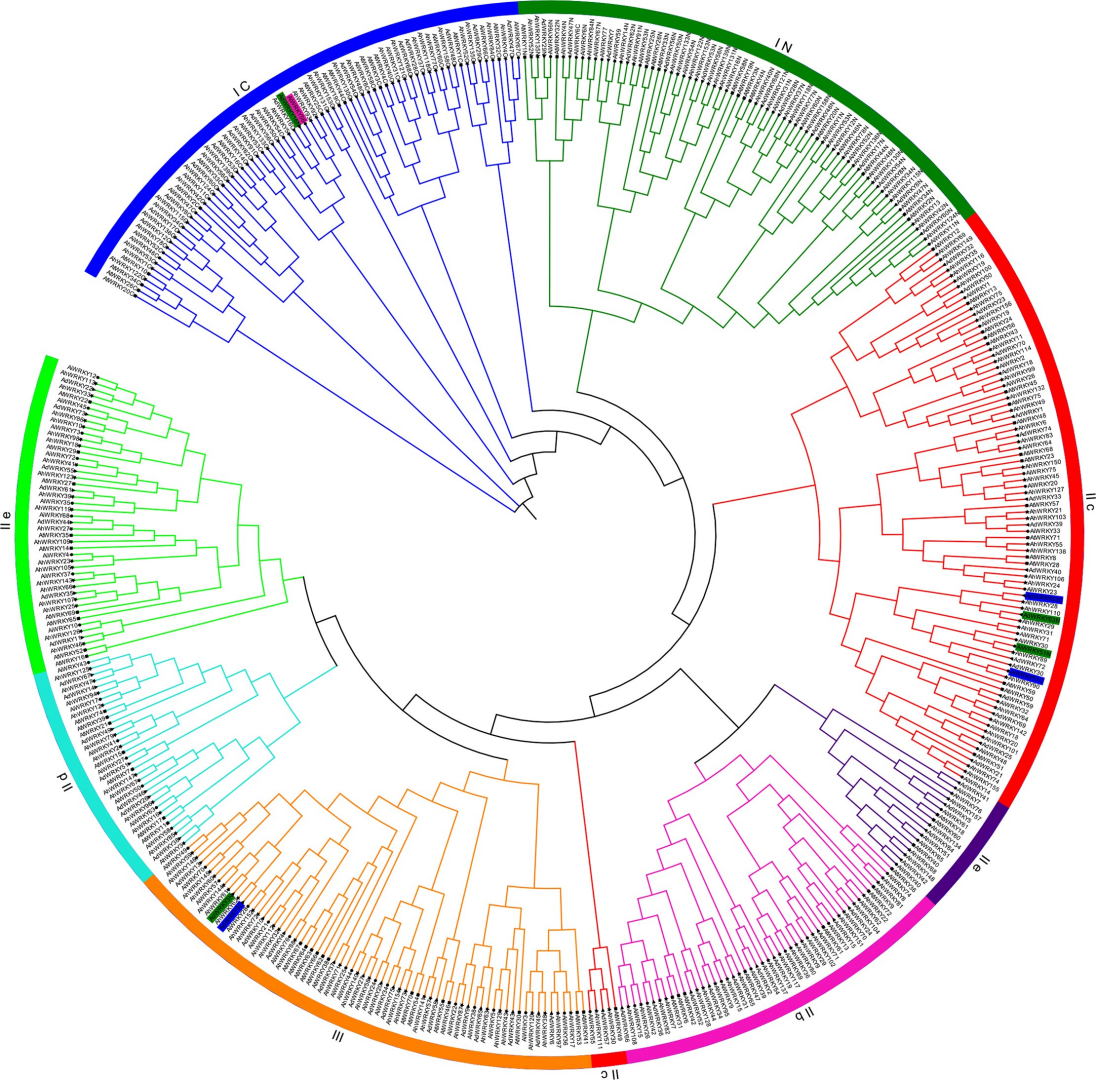
Phylogenetic tree of WRKYs among *Arachis hypogaea*, *Arabidopsis thaliana*, *Arachis duranensis* and *Arachis ipaensis*. The groups (I, II and III) and subgroups (IIa, b, c, d and e) were distinguished in different colors, and the different species were marked by different symbols. Stars represented *WRKY* genes from *Arachis hypogaea*, and squares indicated genes from *Arabidopsis thaliana*. Circles and right triangles indicated *WRKY* genes from *Arachis ipaensis* and *Arachis duranensis*, respectively.

158 AhWRKYs, 72 AtWRKYs, 75 AdWRKYs, and 77 AiWRKYs were used to build the NJ phylogenetic tree (Fig 1). The phylogenetic relationship among AtWRKYs, AdWRKYs and AiWRKYs was consistent with the results of previous studies [8, 18]. Based on results of previous classification [8], the 158 AhWRKYs were grouped into three major clusters and five subgroups; I (33), IIa (6), IIb (22), IIc (38), IId (12), IIe (20), and III (27). However, I-N, I-C, IIb, IIc and III members were found to be nested in other groups or subgroups when the phylogenetic relationship between AdWRKYs and AiWRKYs was analyzed, which is similar to the results obtained in a previous study [18]. The result indicated that the origin of peanut WRKY proteins is still to be clarified. Additionally, the zinc finger motif (C-X_4_-C-X_23_-H-X_1_-C) was identified in AhWRKY71. Interestingly, three (3) WRKYs (AhWRKY71, AhWRKY72, and AhWRKY152) which belonged to group III possessed leucine-rich repeat (LRR) domain that was frequently involved in the formation of protein-protein interactions [10, 18, 54–56]. It is believed that these three (3) proteins may be associated with disease resistance in peanut.

### Gene structure and motif composition of AhWRKYs family

**Fig 2:**
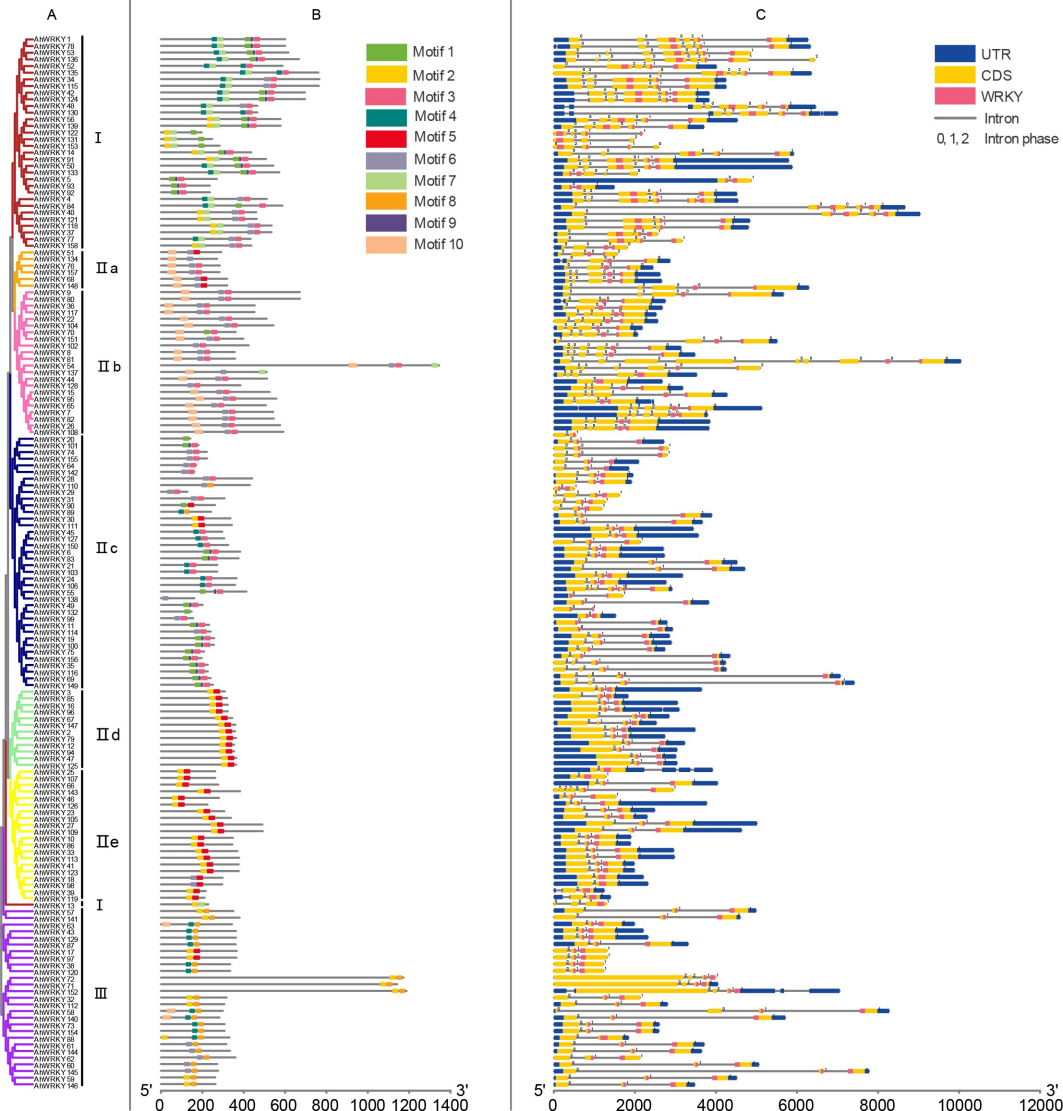
Structure characterization of *AhWRKY* gene family. A Maximum Likelihood (ML) phylogenetic tree of *AhWRKYs*. The various groups were distinguished in different colors. B The conserved motifs of *AhWRKYs*. The 10 motifs were displayed in different-colored boxes. For details of motifs referred to S1 File. C The structures of *AhWRKYs*. The untranslated 5′- and 3′-regions were represented by blue boxes. The yellow boxes and gray lines indicated CDS and introns, respectively. The *WRKY* domains were highlighted by red boxes. The introns phases 0, 1 and 2 were indicated by numbers 0, 1 and 2, respectively.

The exon-intron distribution and motif composition were analyzed to gain more insight into the *AhWRKY* genes based on phylogenetic relationships. Gene structure analysis showed that each of the *AhWRKY* consisted of exons separated by variable numbers of introns. The detailed exon-intron map is shown in Fig 2C. Seventy-one (71) *AhWRKY*s contained two introns and accounted for the maximum proportion (44.9%) of the genes. This was followed by 15 (9.5%), 20 (12.7%), 31 (19.6%), 11 (7.0%), 7 (4.4%) and 3 (1.9%) genes, possessing 1, 3, 4, 5, 6, and 8 introns, respectively. Among them, subgroup IIa genes contained either three (3) or four (4) introns, while most genes in subgroups IIc, IId and IId as well as group III had two (2) introns. The number of introns in group I and subgroup IIb were extremely variable, including 1, 2, 3, 4, 5, 6, 8 and 1, 3, 4, 5, 6, 8, respectively.

Most members of *AhWRKYs* found in the same group had similar exon-intron structures. An intron was located between two complete codons named phase 0, and after the first or second nucleotides in the codon defined phase 1 and 2. Specifically, members of the subgroups IIa and IIb only found phase 0. The same results were observed in pineapple [17]. Phase 1 was widely distributed in other groups with phase 2 having the least distribution.

The conserved motifs were analyzed and assigned numbers from one (1) to ten (10). As shown in Fig 2B, motifs 1, 2, 4, and 6 contained a WRKYGQK sequence. Most AhWRKYs that have been observed to be in the same group or subgroup shared similar motif compositions. For instance, most members of groups IIb contained motifs 3, 6, and 10. Furthermore, subgroup IId and IIe shared motifs 2 and 5 except AhWRKY18 and AhWRKY98, indicating functional similarity among them. Overall, the similar exon-intron structures and conserved motif compositions of the WRKY members in the same group further validated the categorization of AhWRKYs as well as the phylogenetic relationships.

### Chromosome location and gene duplication

**Fig 3:**
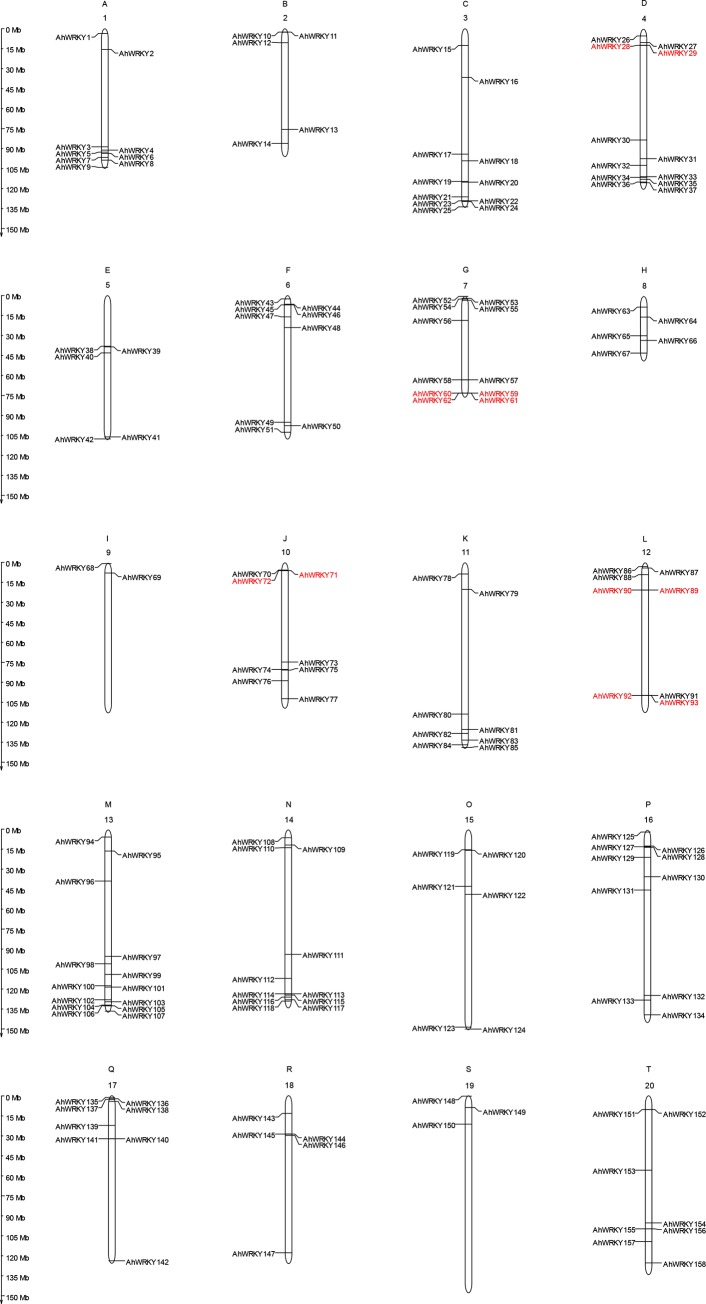
Physical mapping of *AhWRKY* genes on chromosome. The chromosome numbers were indicated at the top of each chromosome. The scale is in megabases (Mb). Tandem duplication genes were marked in red.

The 158 identified *AhWRKY*s were distributed across all the 20 peanut chromosomes (S2 Table; Fig 3) with chromosome 13 containing the highest number (14). On the other hand, chromosomes nine (9) had the least number (2) of *AhWRKYs*. Additionally, 5 chromosomes (10, 11, 12, 17, and 20) had eight (8) *AhWRKYs* whiles chromosomes 1, 3, 4, 6, 7, 14, and 16 harbored relatively more gene members than chromosomes 2, 5, 8, 15, 18, and 19. This is an indication that although the *AhWRKYs* were found on all 20 chromosomes, their distribution across the respective chromosomes was uneven.

**Fig 4:**
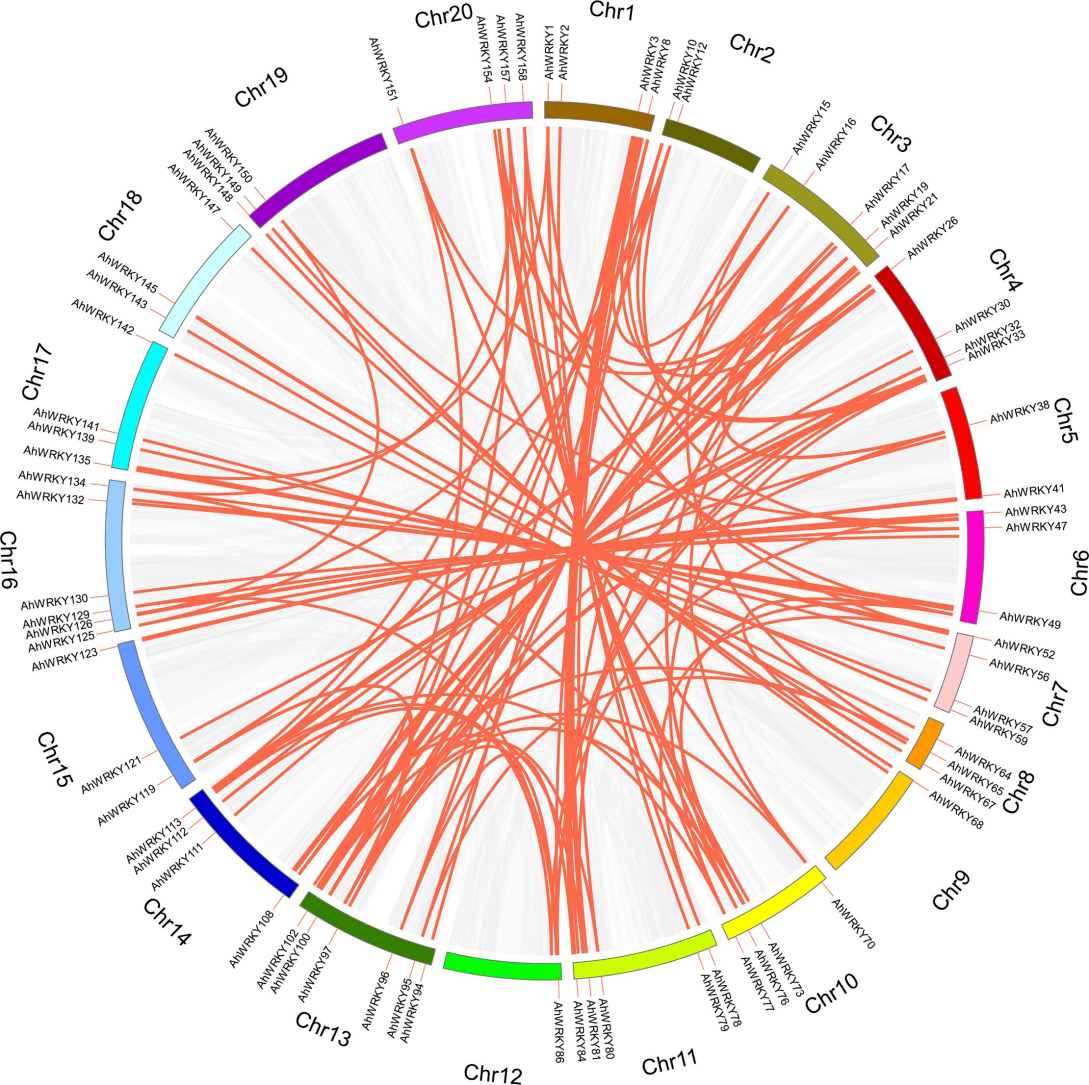
The segmental duplications of *AhWRKY* members. The different color blocks indicated the part of peanut chromosomes. The gray lines indicated all segmental duplications in the peanut genome, and the red lines indicated segmentally duplicated *WRKY* gene pairs.

In the process of evolution, tandem duplication and segmental duplication contribute to the generation of gene families [57]. To explore these events, a chromosomal region within 200 kb containing two or more genes was defined as a gene cluster [58]. According to the defined criteria, 12 tandem duplication genes were found to form six gene clusters (*AhWRKY92*/*AhWRKY93*, *AhWRKY59*/*AhWRKY60*, *AhWRKY89*/*AhWRKY90*, *AhWRKY28*/*AhWRKY29*, *AhWRKY61*/*AhWRKY62* and *AhWRKY71*/*AhWRKY72*). The chromosomes numbered four (4) and ten (10) had one (1) gene clusters, whereas chromosomes numbered seven (7) and twelve (12) contained two (2) gene clusters (Fig 3). On the other hand, 124 segmental duplication events (136 *AhWRKY*s) were found and were distributed on all 20 chromosomes (S3 Table, Fig 4). The results indicated that segmental duplication events were the main driving force in the evolution of *AhWRKY* gene family.

**Fig 5:**
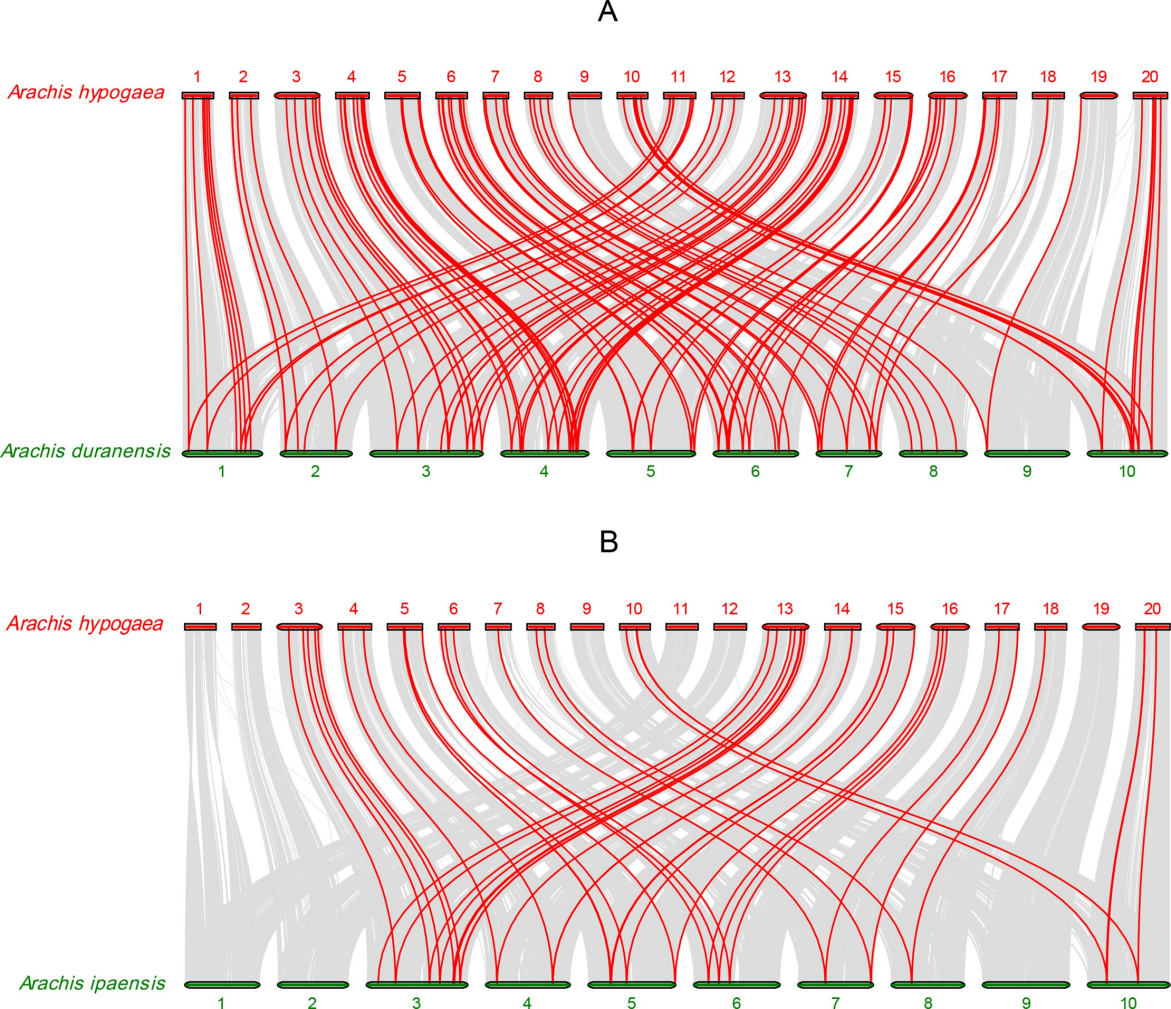
The collinearity analysis of *WRKY* genes between *Arachis hypogaea* and two diploid wild ancestors (*Arachis duranensis* and *Arachis ipaensis*). Gray lines and red lines indicated the all collinear blocks and while the syntenic *WRKY* gene pairs, respectively.

To further infer the phylogenetic mechanisms of peanut WRKY family, two comparative syntenic maps associated with two diploid wild ancestors (*A*. *duranensis* and *A*. *ipaensis*) that have been deduced as the donors of the A and B subgenomes, respectively, were constructed [59–61]. The numbers of collinear gene pairs between *A*. *duranensis* and *A*. *ipaensis* were 110 and 43, respectively (S4 Table). As shown in Fig 5, the collinear gene pairs between the *Arachis hypogaea* and *A*. *duranensis* were randomly distributed on all 20 chromosomes with chromosomes 9, 18 and 19 having the fewest collinear gene pairs. In addition, the collinear gene pairs between *Arachis hypogaea* and *A*. *ipaensis* were less than that of *Arachis hypogaea* and *A*. *duranensis*, and no collinear gene pairs were observed on chromosome 1, 2, 9, 11, 12 and 19. Notably, some collinear gene pairs (32 *AhWRKY*s) were found between *Arachis hypogaea* and *A*. *duranensis*/*A*. *ipaensis*.

### The *cis*-acting elements in promoter regions of AhWRKYs

**Fig 6:**
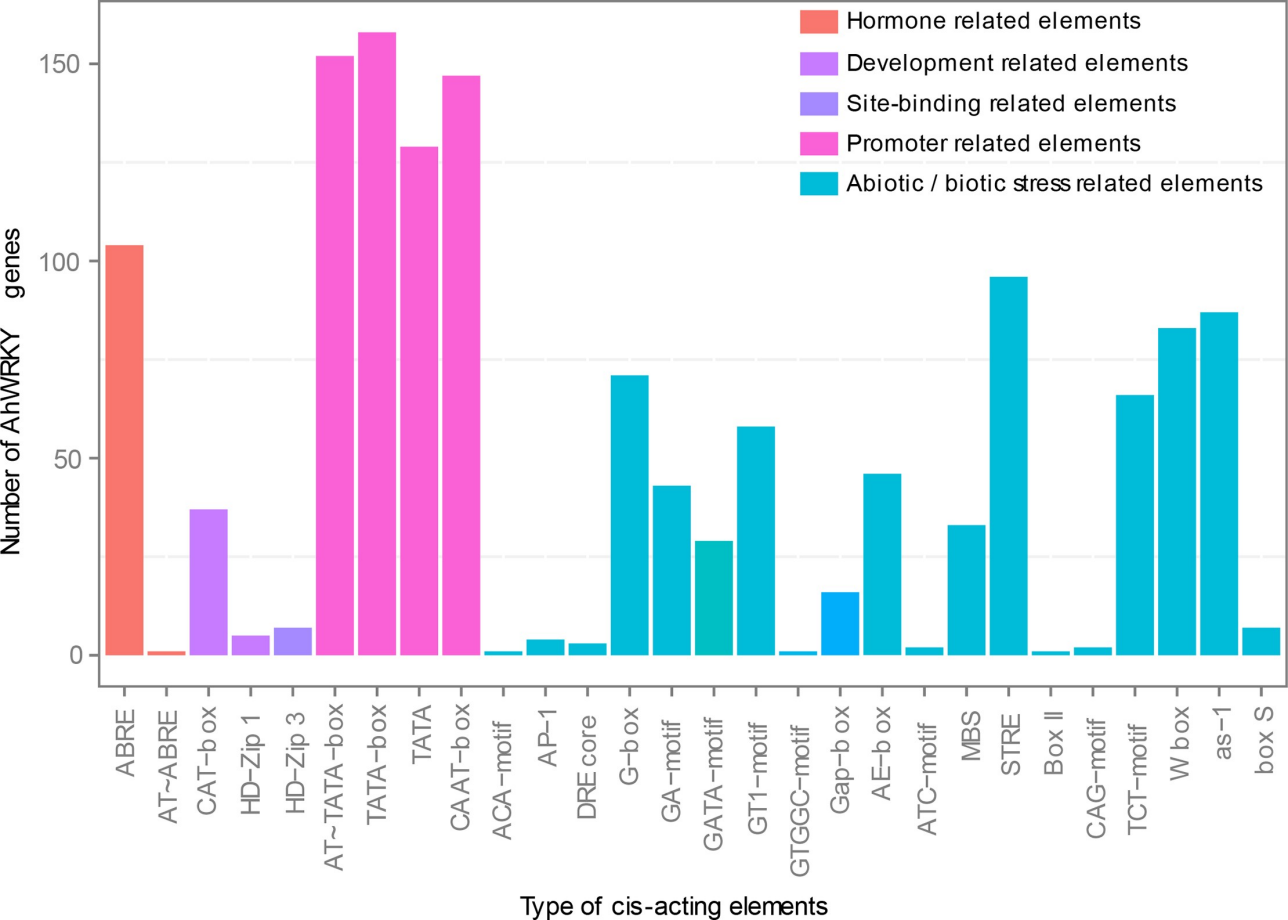
The number of *AhWRKY* genes containing various *cis*-acting elements. *x*-axis and *y*-axis represented the type of *cis*-acting elements and the number of *WRKY* gene family members, respectively.

**Fig 7:**
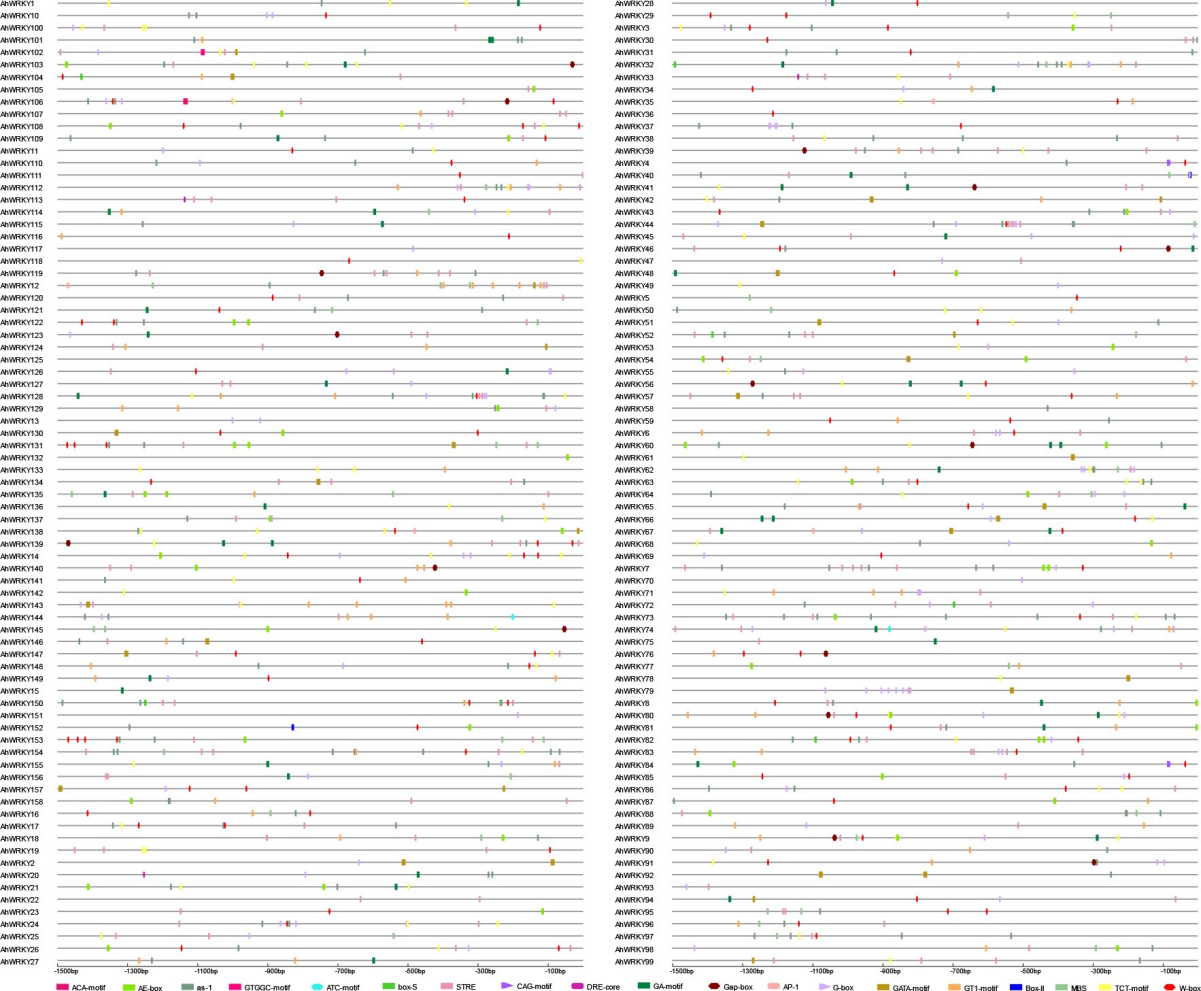
Predicted stress-related *cis*-elements in the *AhWRKY* promoters. The upstream length to the translation start site can be inferred according to the scale at the bottom. Different *cis*-elements were represented by different shapes and colors.

The *cis*-acting elements in the promoter are crucial to gene expression [62]. The 1,500-bp 5’-upstream promoter regions were searched using PlantCARE, and it was found out that the *cis*-acting elements were extremely diverse (S5 Table). The *cis*-elements were classified as stress-related elements, hormone-related elements, development-related elements, promoter related elements and site-binding related elements (Fig 6). The largest number of *cis*-acting elements (19) were related to abiotic/biotic stress responses (Fig 7), and mainly include the MYB binding site involving in drought-inducibility (MBS), the WRKY binding site regulating gene expression (W-box) and the light responsive elements (AE-box, GT1-motif, TCT-motif, GA-motif, G-box, etc.). Interestingly, 83 *AhWRKYs* contained W-box regulated gene expression by binding WRKY, indicating these genes may be auto-regulated or cross-regulated with others. These results indicated that most of *AhWRKY*s might play a vital role in various stress responses.

### Physiological changes in leaves of L422 and L677 under drought treatment

**Fig 8:**
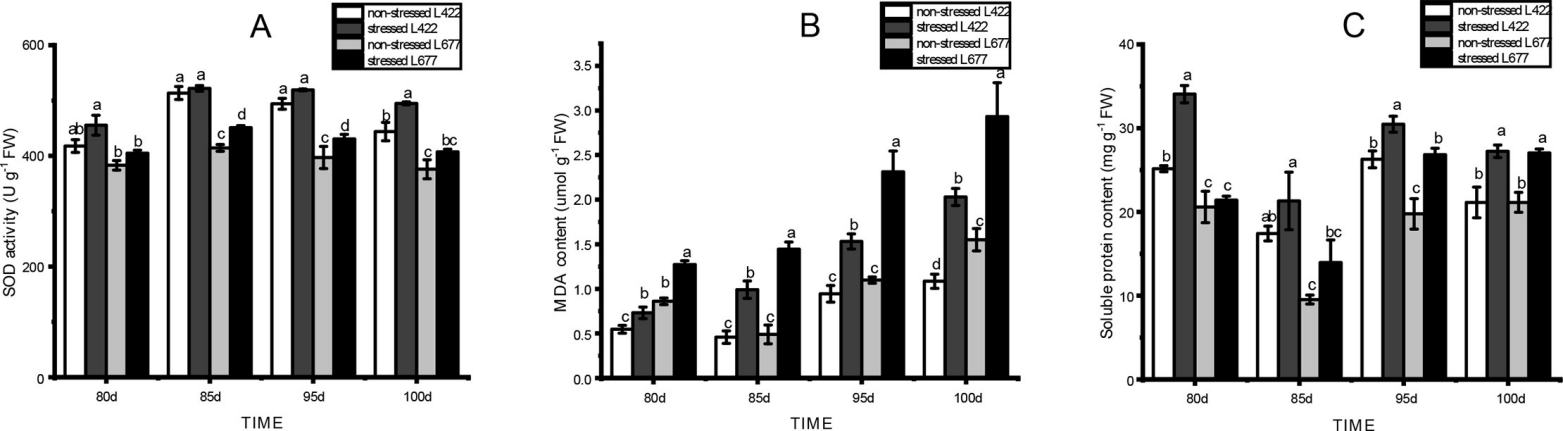
The changes of SOD (A), MDA (B), and Soluble protein content (C) in leaves of drought-tolerant and drought-sensitive varieties under drought stress. Values are mean ± standard error (n = 3). Different letters (a, b, c and d) above line graphs refer to significant difference among treatments (*p*<0.05).

To investigate the peanut response to drought at the pod-setting stage, several phenotype responses induced by drought were studied. As expected, no significant phenotype differences were observed between the two lines under optimum water conditions. However, there were significant differences in the performances of the two lines when exposed to drought stress. The leaves of L677 began to shrivel up, while those of L422 cultivar displayed little or no phenotypic change. As shown in Fig 8A, there were significant differences in SOD activity in both L422 and L677 when the plants were upon exposure to drought stress. The SOD activity was generally higher in L422 than in L677 under stressed condition. The MDA content showed an increasing trend after drought stress in L677 and L422, while the MDA content of L677 was significantly (*p* < 0.05) higher than that of L422 (Fig 8B). The soluble protein content was significantly higher (*p* < 0.05) in L422 than that of in L677 under both stressed and non-stressed conditions (Fig 8C).

### Expression analysis of *AhWRKYs* under drought using RNA-seq

**Fig 9:**
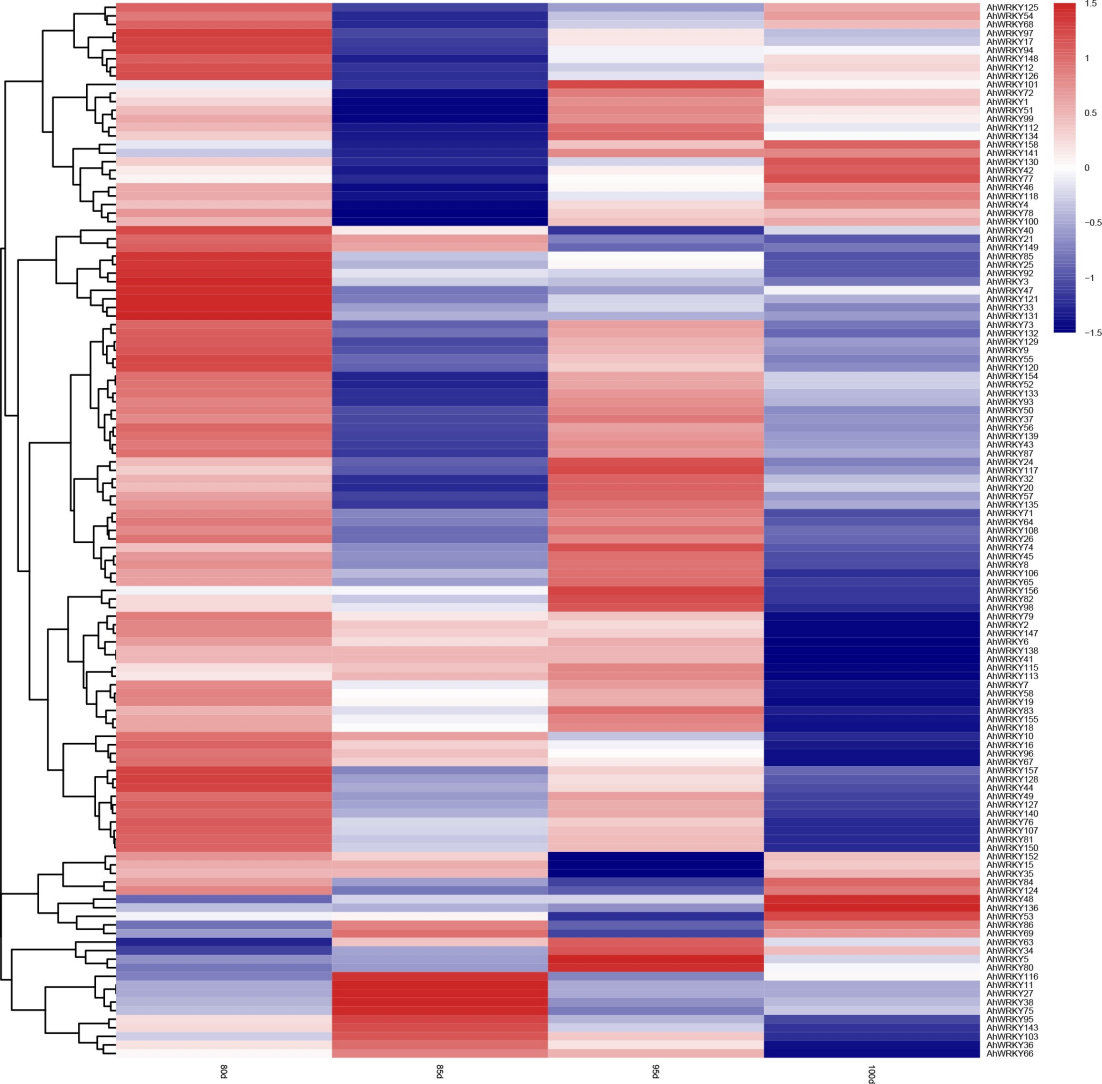
The transcriptomics analysis of the *AhWRKY* genes in response to drought stress. Samples were collected 80, 85, 95 and 100 DAP from the control and drought stress treated peanut. Color scale represented the fold change of drought treatment to control of *AhWRKYs* with normalized log_2_-transformed count value. Red, blue and white indicated high expression, low expression and no expression, respectively.

To investigate specifically the response of *AhWRKY*s to drought stress, expression analysis of the 158 *AhWRKY*s was carried based on transcriptome data obtained under drought (Fig 9 and S6 Table). The relative expression levels were represented as control vs treatment. Genes were considered to be differentially expressed when the value of log_2_ Fold Change was > 1 or < -1 and FDR was less than 0.05 threshold. Twenty (20) out of the 158 *AhWRKY*s were not expressed at all time points, which suggests they might be pseudogenes or may not have been expressed during this period. A total of 4780, 7130, 6240 and 3309 differentially expressed genes were identified at 80, 85, 95 and 100 DAP and include 43, 22, 52 and 14 differentially expressed *WRKY* genes, respectively. Seventy-three (73) differentially expressed *WRKY* genes were influenced by drought stress, of which forty-six (46) were identified in at least two (2) of the four (4) examined time points. Interestingly, two (2) of the genes (*AhWRKY16* and *96*) were initially up-regulated at 80 and 85 DAP while three (3) (*AhWRKY17*, *97* and *148*) were up-regulated at 80 DAP and down-regulated at 85 DAP, respectively. These results suggest that the genes might have a significant role under drought stress at the early pod-setting stage (80–85 DAP). Also, three (3) genes (*AhWRKY18*, *45* and *106*) were up-regulated at 95 DAP although down-regulation was later observed at 100 DAP, which inferred that these three (3) genes responded to drought stress at the latter stage of pod-setting. Moreover, twelve (12) genes (*AhWRKY2*, *6*, *7*, *24*, *49*, *58*, *67*, *68*, *79*, *81*,*132* and *155*) were identified in three of the four examined time points. Overall, the results show *AhWRKY*s has an important role in peanut response to drought stress.

**Fig 10:**
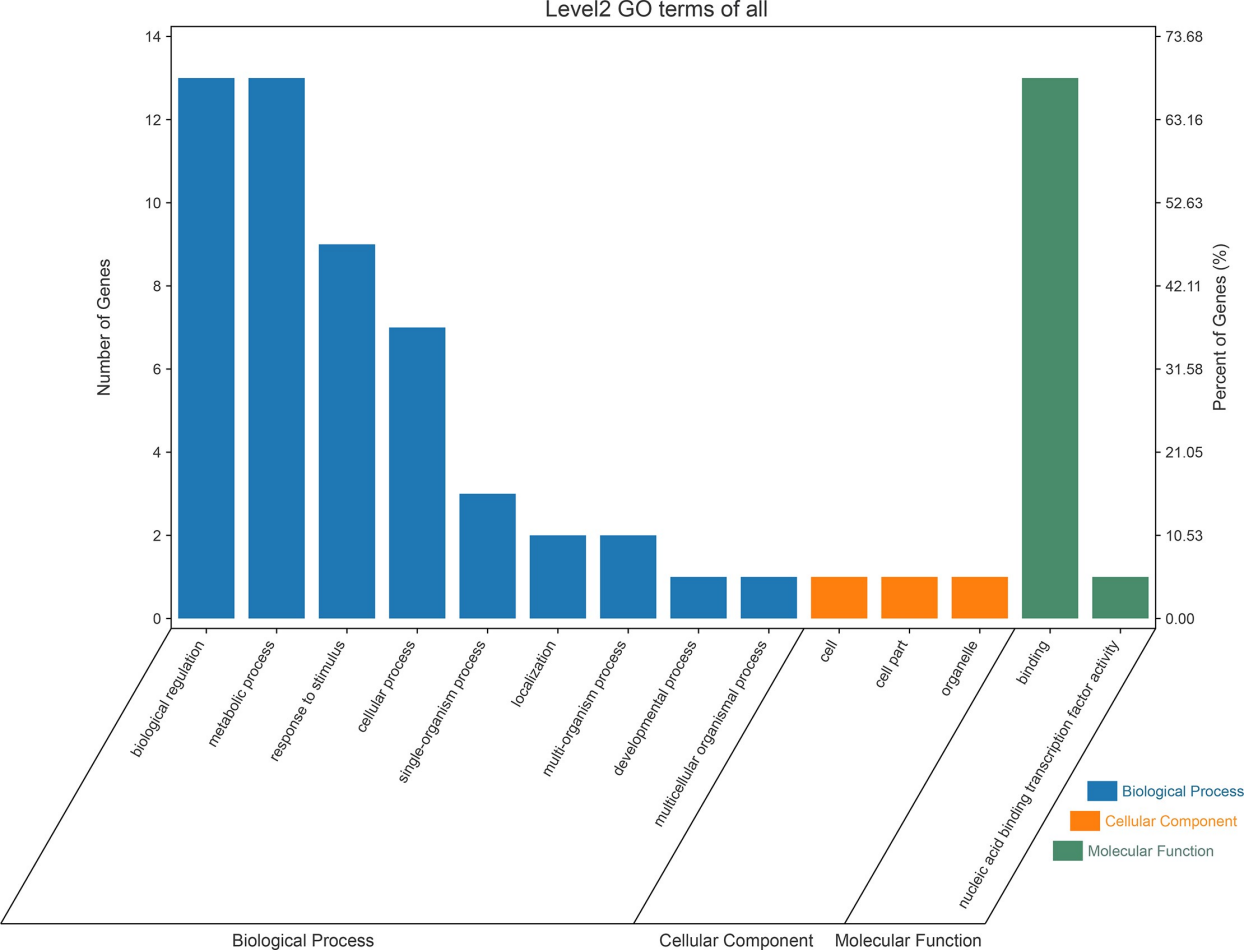
The distribution of GO terms for the differentially expressed *WRKYs*. X-axis represents GO terms; Y-axis on the left is the percentage of differentially expressed *WRKYs* and on the right is the number of differentially expressed *WRKYs*.

To functionally characterize the differentially expressed *WRKY* genes, annotation and pathway analysis were performed (S7 Table). The assigned functions of a total of nineteen (19) differentially expressed *WRKYs* covered a broad range of GO categories (Fig 10). The GO terms for differentially expressed *WRKYs* were mainly related to biological regulation (GO:0065007), metabolic process (GO:0008152), and response to stimulus (GO:0050896). For cellular components, only one (1) *WRKY* gene involved in the cell (GO:0005623), organelle (GO:0043226) and cell part (GO:0044464), respectively. Under molecular functions, the terms only were related to binding (GO:0005488) and nucleic acid binding transcription factor activity (GO:0001071). Genes usually interact with each other to play roles in certain biological functions. Pathway based GO analysis helped to further understand the biological functions of genes. According to the KEGG analysis, the seven (7) and six (6) differentially expressed *WRKY* genes were significantly enriched in plant-pathogen interaction (*AhWRKY56*, *12*, *133*, *50*, 98, *152* and *139*) and plant MAPK signaling pathway (*AhWRKY56*, *12*, *133*, *50*, *98* and *139*), respectively (S7 Table). According to the results of multiple sequence alignment, WRKY152 possessed leucine-rich repeat (LRR) domain, indicating that it might be associated with disease resistance in peanut. Moreover, *WRKY152* was found to be involved in the pathway of plant-pathogen interaction *via* KEGG enrichment analysis. In conclusion, the results demonstrated that *WRKY152* might play an important role in disease resistance processes. Overall, the results of GO and KEGG showed that *AhWRKYs* might not only participated in the drought stress process but also played an important role in other stress-resistant processes.

### Validation of RNA sequencing using qRT-PCR

**Fig 11:**
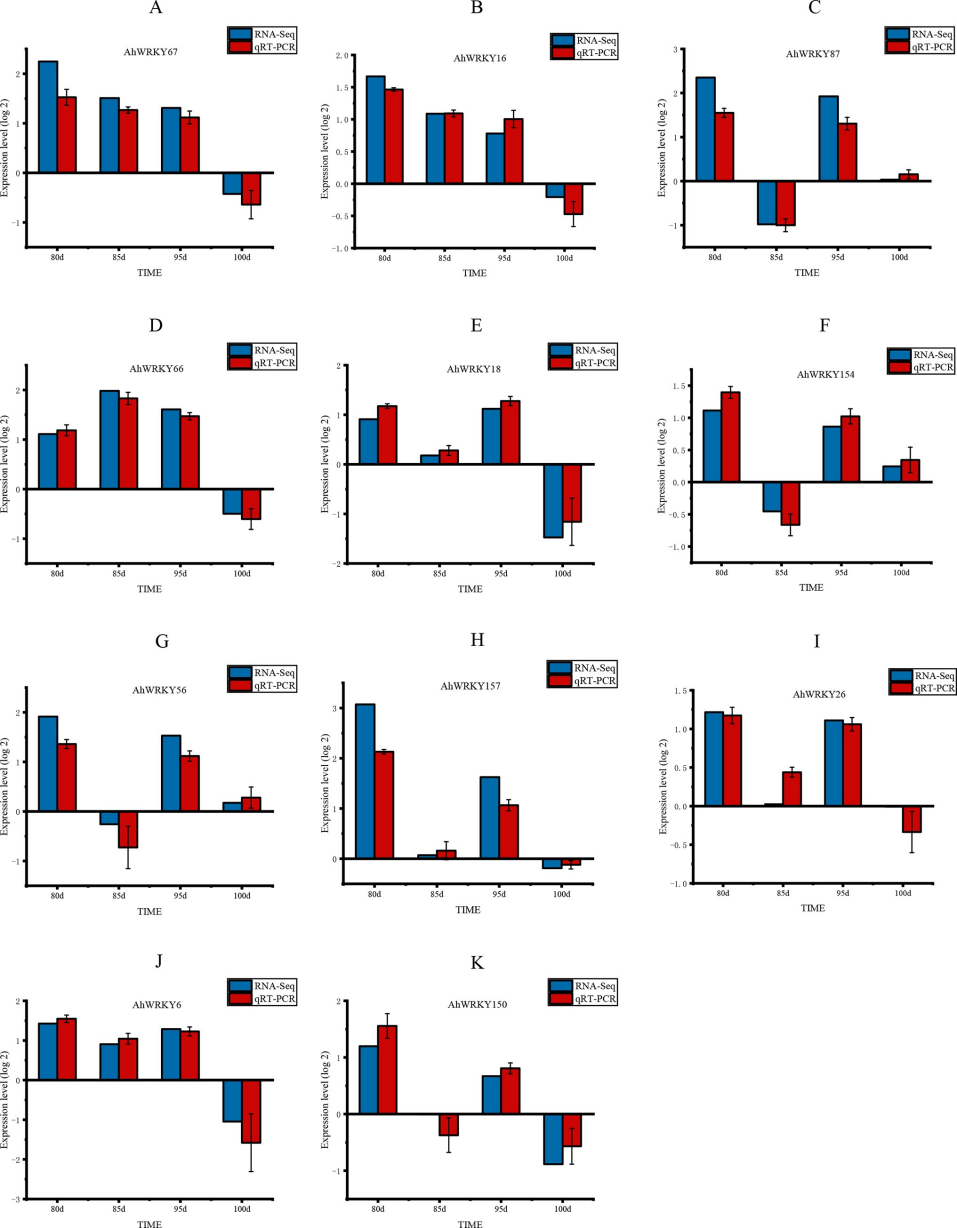
Confirmation of RNA-Seq results by quantitative real-time PCR (qRT-PCR). All genes with negative values of expression level meant that they were down-regulated in response to drought stress.

To validate the expression data obtained from RNA sequencing, 11 differentially expressed *AhWRKY* genes with *cis*-recognition elements noted to have been involved in the abiotic/biotic stress were randomly selected to perform qPCR. The PCR expressions were consistent with the RNA-seq data (Fig 11). A correlation coefficient of 0.89611 was obtained between the fold changes of qRT-PCR and RNA-seq (S2 Fig). The results of the qRT-PCR analysis validated the findings obtained from the RNA-seq data.

## Discussion

The cultivated peanut is an allotetraploid (amphidiploid with 2n = 4x = 40) and it’s mainly cultivated in areas characterized by unstable rainfall pattern. Drought seriously affects the productivity of peanut. The emergence of the genome sequences of *Arachis duranensis* and *Arachis ipaensis* has been an important resource for peanut research on drought resistance [59]. The complete genome sequence data of cultivated peanut has been officially released on PeanutBase (http://www.peanutbase.org/), and it provided the key data to explore functional diversity. WRKY family is a plant-specific transcription factor that participates in plant growth, development and stress responses [6] with 75 *AdWRKYs* and 77 *AiWRKYs* identified in *A*. *duranensis* and *A*. *ipaensis*, respectively [18]. The purpose of this study was to mine potential candidate *AhWRKYs* response to drought stress in peanut which will provide a valuable resource for genetic improvements of agronomically important traits. A total of 158 *AhWRKYs* were identified through genome-wide analysis of the cultivated peanut.

The sequence alignment and phylogenetic tree analysis based on the classification standards for the WRKY family showed the identified 158 AhWRKY proteins from peanut grouped into three main clusters (group I, II and III) with group II proteins further clustering into five subgroups (IIa, IIb, IIc, IId, and IIe) [8]. The subgroup IIc possessed the most members, which were similar to results obtained in many legume crops [12, 14, 18, 63, 64] and was more active in evolution which suggests an important function in legume crops. Majority of the AhWRKYs harbored the highly conserved WRKYGQK motif. However, variants were found in group I and subgroup IIc although most of them were distributed in subgroup IIc. Moreover, variants were mainly observed in *Arachis duranensis* and *Arachis ipaensis* [18]. The observation of variants in subgroup IIc has also been reported in soybean [12], *Arabidopsis* [8] and in pineapple [17]. By inference, WRKYGQK sequence was more likely to mutate in subgroup IIc and hence, members in this subgroup are likely to possess different binding specificities and biological functions by altering their DNA binding affinity [65]. Interestingly, some AhWRKYs were found to contain incomplete zinc finger motifs. However, C2HY was identified in AhWRKY13. These results were consistent with the transcription factor prediction based on the plant transcription factor database (http://planttfdb.cbi.pku.edu.cn/) [66], but the function of variations in zinc finger motif still needs to be further explored. Although AhWRKY5, AhWRKY13, AhWRKY92 and AhWRKY93 were classified into group I, they only possessed one *WRKY* domain while the loss of this domain was found in previous studies [11, 12].

The events of gene duplication have been considered as the main evolutionary driving force of new biological functions [67]. During evolutionary processes, tandem and segmental duplication contribute to the generation of gene family [57]. Among *AdWRKYs* and *AiWRKYs*, four (4) tandem duplication events with eight (8) genes and seven (7) segmental duplication events with 14 genes as well as four (4) tandem duplication events with nine (9) genes and 10 segmental duplication events with 17 genes were found, respectively [18]. In this study, six (6) tandem duplication events with 12 *AhWRKYs* and 124 segmental duplication events with 136 *AhWRKYs* were observed. The number of segmental duplications was significantly (*p*<0.05) more than tandem duplications, which was similar to the results of a previous study on *A*. *duranensis* and *A*. *ipaënsis*. Therefore, it could be deduced that segmental duplication was the main driver for the expansion of *AhWRKYs* family. Also, the comparative synteny mapping of phylogenetic mechanisms of *AhWRKYs* family with *A*. *duranensis* and *A*. *ipaensis* revealed the numbers of collinear gene pairs between *A*. *duranensis* and *A*. *ipaensis* were 110 and 43, respectively. However, only 32 collinear gene pairs were observed between *Arachis hypogaea* and the two diploid species. *A*. *duranensis* and *A*. *ipaensis* were deduced as the donors of the A and B subgenomes, respectively [59–61], and the divergence of the two diploid wild species was estimated to have occurred ~2.16 million years ago [68], which suggested that the 32 collinear gene pairs might have already existed before the divergence of two diploid wild species. In addition, the other *AhWRKYs* could not be mapped to any synteny blocks in this study, but it is not clear whether these genes appeared before or after the hybridization of two diploid wild species doubles.

Drought has a negative effect on physiological processes and hence reduces peanut yield [69]. However, when it is imposed under experimental conditions, it provides valuable clues which enable scientists to derive the function of *AhWRKYs* by comparing the phylogenetic and homologous relationships between *AhWRKYs* and other crop species. For example, *AtWRKY46*, the ortholog gene of *AhWRKY43*, was induced by drought, salt, and oxidative stresses [70]. This suggested that *AhWRKY43* was involved not only in the process of peanut responding to drought stress but also in other physiological processes. *AtWRKY53*, the orthologous gene of *AhWRKY17*, was negatively regulated under drought tolerance by mediating stomatal movement [71]. *AhWRKY43* and *AhWRKY154* were induced by drought treatments, and have been described in their *Arabidopsis* sequence homologues (*AtWRKY46*, *AtWRKY54* and *AtWRKY70*) as playing vital roles in drought responses and brassinosteroid-regulated plant growth [72], while *AhWRKY154* showed no significant differential expression in the current study. The identification of differentially expressed genes under drought stress in *Arachis duranensis* and *Arachis magna* have been released in PeanutBase, which supplied valuable resources for study on drought-related genes in cultivated peanut [73]. *Aradu*.*S7YD6*, the ortholog to *AhWRKY50*, was significantly up-regulated 4.2- and 3.8-fold under drought treatment in *Arachis duranensis* and *Arachis magna*, respectively. *Aradu*.*KG41H*, the ortholog of *AhWRKY43* and *AhWRKY129*, showed a high level of expression (3.9-fold increase) in *Arachis magna*. Based on the homologous relationships, it was deduced that the *AhWRKYs* may play a critical role in the responses to drought stress in cultivated peanut.

## Conclusion

One hundred and fifty-eight *AhWRKYs*, distributing across 20 chromosomes of cultivated peanut, were identified and grouped into three main groups based on the sequence alignment and phylogenetic tree analysis. Gene duplication analysis indicated that segmental duplication events were the main driving force in the evolution of *WRKY* gene family in peanut. The synteny analysis of *Arachis hypogaea* and two diploid wild ancestors (*A*. *duranensis* and *A*. *ipaensis*) provided helpful information for the evolutionary characteristics of *AhWRKYs*. The functional annotations, *cis*-acting elements and expression analysis revealed that most *AhWRKYs* were involved in response to drought stress. These results will provide a valuable resource for further investigation of peanut drought tolerance characteristics.

## Supporting information

S1 Fig Multiple sequence alignment of AhWRKYs and selected AtWRKY domain amino acid sequences. The highly conserved WRKYGQK heptapeptide, and the amino acids forming the zinc-finger motif were covered in blue boxes, while the mutated amino acids were marked in red.
(PDF)

S2 Fig Correlation between RNA sequencing and qRT-PCR for the eleven randomly selected differentially expressed *AhWRKY* genes. (PDF)

S1 File Analysis and distribution of conserved motifs in peanut *WRKY* proteins. (PDF)

S1 Table Sequences of the primers used in this study. (XLSX)

S2 Table List of the 158 *AhWRKY* genes identified in this study. (XLSX)

S3 Table Segmentally and tandemly duplicated *AhWRKY* gene pairs. (XLSX)

S4 Table Orthologous relationships between *Arachis hypogaea* and two diploid wild species. (XLSX)

S5 Table List of the type of *cis*-acting elements. (XLSX)

S6 Table RNA-seq data of 158 *AhWRKY* genes that were used in this study. (XLSX)

S7 Table Functional annotation of the differentially expressed *AhWRKY* genes under drought stress. (XLSX)

## References

[1] Rushton PJ, Somssich IE, Ringler P, Shen QJ. WRKY transcription factors. Trends in Plant Science. 2010;15(5):247–258. 10.1016/j.tplants.2010.02.006 20304701

[2] Agarwal P, Reddy M, Chikara J. WRKY: its structure, evolutionary relationship, DNA-binding selectivity, role in stress tolerance and development of plants. Molecular Biology Reports. 2011;38(6):3883–3896. 10.1007/s11033-010-0504-5 21107718

[3] Eulgem T, Somssich IE. Networks of WRKY transcription factors in defense signaling. Current Opinion in Plant Biology. 2007;10(4):366–371. 10.1016/j.pbi.2007.04.020 17644023

[4] Ishihama N, Yoshioka H. Post-translational regulation of WRKY transcription factors in plant immunity. Current Opinion in Plant Biology. 2012;15(4):431–437. 10.1016/j.pbi.2012.02.003 22425194

[5] Jiang J, Ma S, Ye N, Jiang M, Cao J, Zhang J. WRKY transcription factors in plant responses to stresses. Journal of Integrative Plant Biology. 2017;59(2):86–101. 10.1111/jipb.12513 27995748

[6] Ülker B, Somssich IE. WRKY transcription factors: from DNA binding towards biological function. Current Opinion in Plant Biology. 2004;7(5):491–498. 10.1016/j.pbi.2004.07.012 15337090

[7] Viana VE, Busanello C, da Maia LC, Pegoraro C, Costa de Oliveira A. Activation of rice WRKY transcription factors: an army of stress fighting soldiers? Current Opinion in Plant Biology. 2018;45:268–275. 10.1016/j.pbi.2018.07.007 30060992

[8] Eulgem T, Rushton PJ, Robatzek S, Somssich IE. The WRKY superfamily of plant transcription factors. Trends in Plant Science. 2000;5(5):199–206. 10.1016/s1360-1385(00)01600-9 10785665

[9] Ishiguro S, Nakamura K. Characterization of a cDNA encoding a novel DNA-binding protein, SPF1, that recognizes SP8 sequences in the 5' upstream regions of genes coding for sporamin and beta-amylase from sweet potato. Molecular and General Genetics. 1994;244(6):563–571. 10.1007/bf00282746 7969025

[10] Kalde M, Barth M, Somssich IE, Lippok B. Members of the *Arabidopsis* WRKY group III transcription factors are part of different plant defense signaling pathways. Molecular Plant-Microbe Interactions. 2003;16(4):295–305. 10.1094/MPMI.2003.16.4.295 12744458

[11] Ross CA, Liu Y, Shen QJ. The WRKY gene family in rice (Oryza sativa). Journal of Integrative Plant Biology. 2007;49(6):827–842. 10.1111/j.1744-7909.2007.00504.x

[12] Yin G, Xu H, Xiao S, Qin Y, Li Y, Yan Y, et al The large soybean (*Glycine max*) WRKY TF family expanded by segmental duplication events and subsequent divergent selection among subgroups. BMC Plant Biology. 2013;13:148 10.1186/1471-2229-13-148 24088323 PMC3850935

[13] Cai C, Niu E, Du H, Zhao L, Feng Y, Guo W. Genome-wide analysis of the WRKY transcription factor gene family in *Gossypium raimondii* and the expression of orthologs in cultivated tetraploid cotton. Crop Journal. 2014;2(2–3):87–101. 10.1016/j.cj.2014.03.001

[14] Song H, Wang P, Nan Z, Wang X. The WRKY transcription factor genes in *Lotus japonicus*. International Journal of Genomics. 2014;2014:420128 10.1155/2014/420128 24745006 PMC3976811

[15] Okay S, Derelli E, Unver T. Transcriptome-wide identification of bread wheat WRKY transcription factors in response to drought stress. Molecular Genetics and Genomics. 2014;289(5):765–781. 10.1007/s00438-014-0849-x 24748053

[16] Wang M, Vannozzi A, Wang G, Liang Y-H, Tornielli GB, Zenoni S, et al Genome and transcriptome analysis of the grapevine (*Vitis vinifera* L.) WRKY gene family. Horticulture Research. 2014;1:14016 10.1038/hortres.2014.16 26504535 PMC4596322

[17] Xie T, Chen C, Li C, Liu J, Liu C, He Y. Genome-wide investigation of WRKY gene family in pineapple: evolution and expression profiles during development and stress. BMC Genomics. 2018;19(1):490 10.1186/s12864-018-4880-x 29940851 PMC6019807

[18] Song H, Wang P, Lin JY, Zhao C, Bi Y, Wang X. Genome-wide identification and characterization of *WRKY* gene family in peanut. Frontiers in Plant Science. 2016;7:534 10.3389/fpls.2016.00534 27200012 PMC4845656

[19] Luan Q, Chen C, Liu M, Li Q, Wang L, Ren Z. CsWRKY50 mediates defense responses to *Pseudoperonospora cubensis* infection in *Cucumis sativus*. Plant Science. 2019;279:59–69. 10.1016/j.plantsci.2018.11.002 30709494

[20] Liu X, Bai X, Wang X, Chu C. OsWRKY71, a rice transcription factor, is involved in rice defense response. Journal of Plant Physiology. 2007;164(8):969–979. 10.1016/j.jplph.2006.07.006 16919842

[21] Tian X, Li X, Zhou W, Ren Y, Wang Z, Liu Z, et al Transcription factor OsWRKY53 positively regulates brassinosteroid signaling and plant architecture. Plant Physiology. 2017;175(3):1337–1349. 10.1104/pp.17.00946 28894020 PMC5664471

[22] Zhang C-Q, Xu Y, Lu Y, Yu H-X, Gu M-H, Liu Q-Q. The WRKY transcription factor OsWRKY78 regulates stem elongation and seed development in rice. Planta. 2011;234(3):541–554. 10.1007/s00425-011-1423-y 21547461

[23] Chen L, Zhang L, Li D, Wang F, Yu D. WRKY8 transcription factor functions in the TMV-cg defense response by mediating both abscisic acid and ethylene signaling in *Arabidopsis*. Proceedings of the National Academy of Sciences of the United States of America. 2013;110(21):E1963–E1971. 10.1073/pnas.1221347110 23650359 PMC3666684

[24] Devaiah BN, Karthikeyan AS, Raghothama KG. WRKY75 transcription factor is a modulator of phosphate acquisition and root development in *Arabidopsis*. Plant Physiology. 2007;143(4):1789–1801. 10.1104/pp.106.093971 17322336 PMC1851818

[25] Robatzek S, Somssich IE. A new member of the *Arabidopsis* WRKY transcription factor family, AtWRKY6, is associated with both senescence- and defence-related processes. Plant Journal. 2001;28(2):123–133. 10.1046/j.1365-313x.2001.01131.x 11722756

[26] Sheng Y, Yan X, Huang Y, Han Y, Zhang C, Ren Y, et al The WRKY transcription factor, WRKY13, activates *PDR8* expression to positively regulate cadmium tolerance in *Arabidopsis*. Plant, Cell & Environment. 2019;42(3):891–903. 10.1111/pce.13457 30311662

[27] Zhu D, Hou L, Xiao P, Guo Y, Deyholos MK, Liu X. VvWRKY30, a grape WRKY transcription factor, plays a positive regulatory role under salinity stress. Plant Science. 2019;280:132–142. 10.1016/j.plantsci.2018.03.018 30823991

[28] Gao H, Wang Y, Xu P, Zhang Z. Overexpression of a WRKY transcription factor *TaWRKY2* enhances drought stress tolerance in transgenic wheat. Frontiers in Plant Science. 2018;9:997 10.3389/fpls.2018.00997 30131813 PMC6090177

[29] Lagacé M, Matton DP. Characterization of a WRKY transcription factor expressed in late torpedo-stage embryos of *Solanum chacoense*. Planta. 2004;219(1):185–189. 10.1007/s00425-004-1253-2 15045588

[30] Shao HB, Chu LY, Jaleel CA, Manivannan P, Panneerselvam R, Shao MA. Understanding water deficit stress-induced changes in the basic metabolism of higher plants—biotechnologically and sustainably improving agriculture and the ecoenvironment in arid regions of the globe. Critical Reviews in Biotechnology. 2009;29(2):131–151. 10.1080/07388550902869792 19412828

[31] Ramachandra Reddy A, Chaitanya KV, Vivekanandan M. Drought-induced responses of photosynthesis and antioxidant metabolism in higher plants. Journal of Plant Physiology. 2004;161(11):1189–1202. 10.1016/j.jplph.2004.01.013 15602811

[32] Chen Y, Liu ZH, Feng L, Zheng Y, Li DD, Li XB. Genome-wide functional analysis of cotton (*Gossypium hirsutum*) in response to drought. PloS One. 2013;8(11):e80879 10.1371/journal.pone.0080879 24260499 PMC3832458

[33] Budak H, Hussain B, Khan Z, Ozturk NZ, Ullah N. From genetics to functional genomics: improvement in drought signaling and tolerance in wheat. Front Plant Sci. 2015;6:1012 10.3389/fpls.2015.01012 26635838 PMC4652017

[34] Kumar P, Rouphael Y, Cardarelli M, Colla G. Vegetable Grafting as a Tool to Improve Drought Resistance and Water Use Efficiency. Front Plant Sci. 2017;8:1130 10.3389/fpls.2017.01130 28713405 PMC5492162

[35] Bertioli DJ, Jenkins J, Clevenger J, Dudchenko O, Gao D, Seijo G, et al The genome sequence of segmental allotetraploid peanut *Arachis hypogaea*. Nature Genetics. 2019;51(5):877–884. 10.1038/s41588-019-0405-z 31043755

[36] Chou KC, Shen HB. Plant-mPLoc: a top-down strategy to augment the power for predicting plant protein subcellular localization. PloS One. 2010;5(6):e11335 10.1371/journal.pone.0011335 20596258 PMC2893129

[37] Larkin MA, Blackshields G, Brown N, Chenna R, McGettigan PA, McWilliam H, et al Clustal W and Clustal X version 2.0. Bioinformatics. 2007;23(21):2947–2948. 10.1093/bioinformatics/btm404 17846036

[38] Kumar S, Stecher G, Tamura K. MEGA7: molecular evolutionary genetics analysis version 7.0 for bigger datasets. Molecular Biology and Evolution. 2016;33(7):1870–1874. 10.1093/molbev/msw054 27004904 PMC8210823

[39] Hu B, Jin J, Guo A-Y, Zhang H, Luo J, Gao G. GSDS 2.0: an upgraded gene feature visualization server. Bioinformatics. 2014;31(8):1296–1297. 10.1093/bioinformatics/btu817 25504850 PMC4393523

[40] Lescot M, Déhais P, Thijs G, Marchal K, Moreau Y, Van de Peer Y, et al PlantCARE, a database of plant *cis*-acting regulatory elements and a portal to tools for *in silico* analysis of promoter sequences. Nucleic Acids Research. 2002;30(1):325–327. 10.1093/nar/30.1.325 11752327 PMC99092

[41] Bailey TL, Williams N, Misleh C, Li WW. MEME: discovering and analyzing DNA and protein sequence motifs. Nucleic Acids Research. 2006;34:W369–W373. 10.1093/nar/gkl198 16845028 PMC1538909

[42] Wang Y, Tang H, Debarry JD, Tan X, Li J, Wang X, et al MCScanX: a toolkit for detection and evolutionary analysis of gene synteny and collinearity. Nucleic Acids Research. 2012;40(7):e49 10.1093/nar/gkr1293 22217600 PMC3326336

[43] Krzywinski M, Schein J, Birol I, Connors J, Gascoyne R, Horsman D, et al Circos: an information aesthetic for comparative genomics. Genome Research. 2009;19(9):1639–1645. 10.1101/gr.092759.109 19541911 PMC2752132

[44] Liu JJ, Wei Z, Li JH. Effects of copper on leaf membrane structure and root activity of maize seedling. Botanical Studies. 2014;55 10.1186/s40529-014-0047-5 PMC5432969 28510936

[45] Jiang MY, Zhang JH. Water stress-induced abscisic acid accumulation triggers the increased generation of reactive oxygen species and up-regulates the activities of antioxidant enzymes in maize leaves. Journal of Experimental Botany. 2002;53(379):2401–2410. 10.1093/jxb/erf090 12432032

[46] Bradford MM. A rapid and sensitive method for the quantitation of microgram quantities of protein utilizing the principle of protein-dye binding. Analytical Biochemistry. 1976;72:248–254. 10.1006/abio.1976.9999 942051

[47] Kim D, Pertea G, Trapnell C, Pimentel H, Kelley R, Salzberg SL. TopHat2: accurate alignment of transcriptomes in the presence of insertions, deletions and gene fusions. Genome Biology. 2013;14(4). 10.1186/gb-2013-14-4-r36 PMC4053844 23618408

[48] Mortazavi A, Williams BA, McCue K, Schaeffer L, Wold B. Mapping and quantifying mammalian transcriptomes by RNA-Seq. Nature Methods. 2008;5(7):621–628. 10.1038/nmeth.1226 18516045 PMC13303166

[49] Pertea M, Pertea GM, Antonescu CM, Chang TC, Mendell JT, Salzberg SL. StringTie enables improved reconstruction of a transcriptome from RNA-seq reads. Nature Biotechnology. 2015;33(3):290–+. 10.1038/nbt.3122 25690850 PMC4643835

[50] Leng N, Dawson JA, Thomson JA, Ruotti V, Rissman AI, Smits BMG, et al EBSeq: an empirical Bayes hierarchical model for inference in RNA-seq experiments (vol 29, pg 1035, 2013). Bioinformatics. 2013;29(16):2073–2073. 10.1093/bioinformatics/btt337 PMC3624807 23428641

[51] Benjamini Y, Hochberg Y. Controlling the False Discovery Rate: a Practical and Powerful Approach to Multiple Testing. Journal of the Royal Statistical Society Series B-Statistical Methodology. 1995;57(1):289–300. 10.1111/j.2517-6161.1995.tb02031.x

[52] Reddy DS, Bhatnagar-Mathur P, Cindhuri KS, Sharma KK. Evaluation and validation of reference genes for normalization of quantitative real-time PCR based gene expression studies in peanut. PloS One. 2013;8(10):e78555 10.1371/journal.pone.0078555 24167633 PMC3805511

[53] Livak KJ, Schmittgen TD. Analysis of relative gene expression data using real-time quantitative PCR and the 2^-△△CT^ method. Methods. 2001;25(4):402–408. 10.1006/meth.2001.1262 11846609

[54] Kobe B, Kajava AV. The leucine-rich repeat as a protein recognition motif. Current Opinion in Structural Biology. 2001;11(6):725–732. 10.1016/s0959-440x(01)00266-4 11751054

[55] Rinerson CI, Rabara RC, Tripathi P, Shen QJ, Rushton PJ. The evolution of WRKY transcription factors. BMC Plant Biology. 2015;15:66 10.1186/s12870-015-0456-y 25849216 PMC4350883

[56] Song H, Guo Z, Chen T, Sun J, Yang G. Genome-wide identification of LRR-containing sequences and the response of these sequences to nematode infection in *Arachis duranensis*. BMC Plant Biology. 2018;18(1):279 10.1186/s12870-018-1508-x 30424729 PMC6234637

[57] Cannon SB, Mitra A, Baumgarten A, Young ND, May G. The roles of segmental and tandem gene duplication in the evolution of large gene families in *Arabidopsis thaliana*. BMC Plant Biology. 2004;4:10 10.1186/1471-2229-4-10 15171794 PMC446195

[58] Holub EB. The arms race is ancient history in *Arabidopsis*, the wildflower. Nature Reviews: Genetics. 2001;2(7):516–527. 10.1038/35080508 11433358

[59] Bertioli DJ, Cannon SB, Froenicke L, Huang G, Farmer AD, Cannon EK, et al The genome sequences of *Arachis duranensis* and *Arachis ipaensis*, the diploid ancestors of cultivated peanut. Nature Genetics. 2016;48(4):438–446. 10.1038/ng.3517 26901068

[60] Moretzsohn MC, Gouvea EG, Inglis PW, Leal-Bertioli SC, Valls JF, Bertioli DJ. A study of the relationships of cultivated peanut (*Arachis hypogaea*) and its most closely related wild species using intron sequences and microsatellite markers. Annals of Botany. 2013;111(1):113–126. 10.1093/aob/mcs237 23131301 PMC3523650

[61] Seijo G, Lavia GI, Fernández A, Krapovickas A, Ducasse DA, Bertioli DJ, et al Genomic relationships between the cultivated peanut (*Arachis hypogaea*, Leguminosae) and its close relatives revealed by double GISH. American Journal of Botany. 2007;94(12):1963–1971. 10.3732/ajb.94.12.1963 21636391

[62] Rombauts S, Déhais P, Van Montagu M, Rouzé P. PlantCARE, a plant *cis*-acting regulatory element database. Nucleic Acids Research. 1999;27(1):295–296. 10.1093/nar/27.1.295 9847207 PMC148162

[63] Song H, Sun W, Yang G, Sun J. WRKY transcription factors in legumes. BMC Plant Biology. 2018;18(1):243 10.1186/s12870-018-1467-2 30332991 PMC6192229

[64] Srivastava R, Kumar S, Kobayashi Y, Kusunoki K, Tripathi P, Kobayashi Y, et al Comparative genome-wide analysis of WRKY transcription factors in two Asian legume crops: Adzuki bean and Mung bean. Scientific Reports. 2018;8(1):16971 10.1038/s41598-018-34920-8 30451872 PMC6243003

[65] Zhou QY, Tian AG, Zou HF, Xie ZM, Lei G, Huang J, et al Soybean WRKY-type transcription factor genes, *GmWRKY13*, *GmWRKY21*, and *GmWRKY54*, confer differential tolerance to abiotic stresses in transgenic *Arabidopsis* plants. Plant Biotechnology Journal. 2008;6(5):486–503. 10.1111/j.1467-7652.2008.00336.x 18384508

[66] Jin J, Tian F, Yang DC, Meng YQ, Kong L, Luo J, et al PlantTFDB 4.0: toward a central hub for transcription factors and regulatory interactions in plants. Nucleic Acids Research. 2017;45(D1):D1040–D1045. 10.1093/nar/gkw982 27924042 PMC5210657

[67] Kondrashov FA, Rogozin IB, Wolf YI, Koonin EV. Selection in the evolution of gene duplications. Genome Biology. 2002;3(2):RESEARCH0008. 10.1186/gb-2002-3-2-research0008 PMC65685 11864370

[68] Chen X, Lu Q, Liu H, Zhang J, Hong Y, Lan H, et al Sequencing of cultivated peanut, *Arachis hypogaea*, yields insights into genome evolution and oil improvement. Molecular Plant. 2019 10.1016/j.molp.2019.03.005 30902685

[69] Arruda IMa, Modacirino V, Buratto JS, Ferreira JM, Arruda IMa, Modacirino V, et al Growth and yield of peanut cultivars and breeding lines under water deficit. Pesquisa Agropecuária Tropical. 2015;45:146–154. 10.1590/1983-40632015v4529652

[70] Ding ZJ, Yan JY, Xu XY, Yu DQ, Li GX, Zhang SQ, et al Transcription factor WRKY 46 regulates osmotic stress responses and stomatal movement independently in Arabidopsis. Plant Journal. 2014;79(1):13–27. 10.1111/tpj.12538 24773321

[71] Sun Y, Yu D. Activated expression of AtWRKY53 negatively regulates drought tolerance by mediating stomatal movement. Plant Cell Reports. 2015;34(8):1295–1306. 10.1007/s00299-015-1787-8 25861729

[72] Chen J, Nolan TM, Ye H, Zhang M, Tong H, Xin P, et al Arabidopsis WRKY46, WRKY54, and WRKY70 transcription factors are involved in brassinosteroid-regulated plant growth and drought responses. Plant Cell. 2017;29(6):1425–1439. 10.1105/tpc.17.00364 28576847 PMC5502465

[73] Brasileiro AC, Morgante CV, Araujo AC, Leal-Bertioli SC, Silva AK, Martins AC, et al Transcriptome profiling of wild arachis from water-limited environments uncovers drought tolerance candidate genes. Plant Molecular Biology Reporter. 2015;33:1876–1892. 10.1007/s11105-015-0882-x 26752807 PMC4695501

