# Supplementary material for: Identification and expression analysis of WRKY gene family under drought stress in peanut (Arachis hypogaea L.)
Source: PLoS One. 2020 Apr 9;15(4):e0231396. doi: 10.1371/journal.pone.0231396 (PMC7144997; doi:10.1371/journal.pone.0231396)
Supplement: S1 Fig — The highly conserved WRKYGQK heptapeptide, and the amino acids forming the zinc-finger motif were covered in blue boxes, while the mutated amino acids were marked in red. (PDF) [file pone.0231396.s001.pdf]

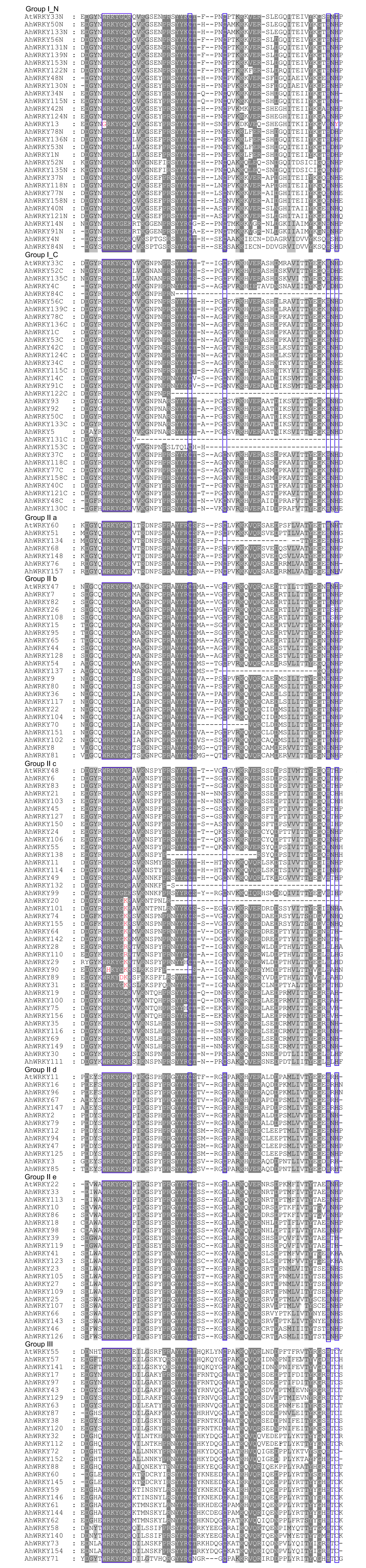
Group I_N
Group I_C
Group II a
Group II b
Group II c
Group II d
Group II e
Group III
