# Supplementary figures and images for: Identification and expression analysis of WRKY gene family under drought stress in peanut (Arachis hypogaea L.)
Source: PLoS One. 2020 Apr 9;15(4):e0231396. doi: 10.1371/journal.pone.0231396 (PMC7144997; doi:10.1371/journal.pone.0231396)

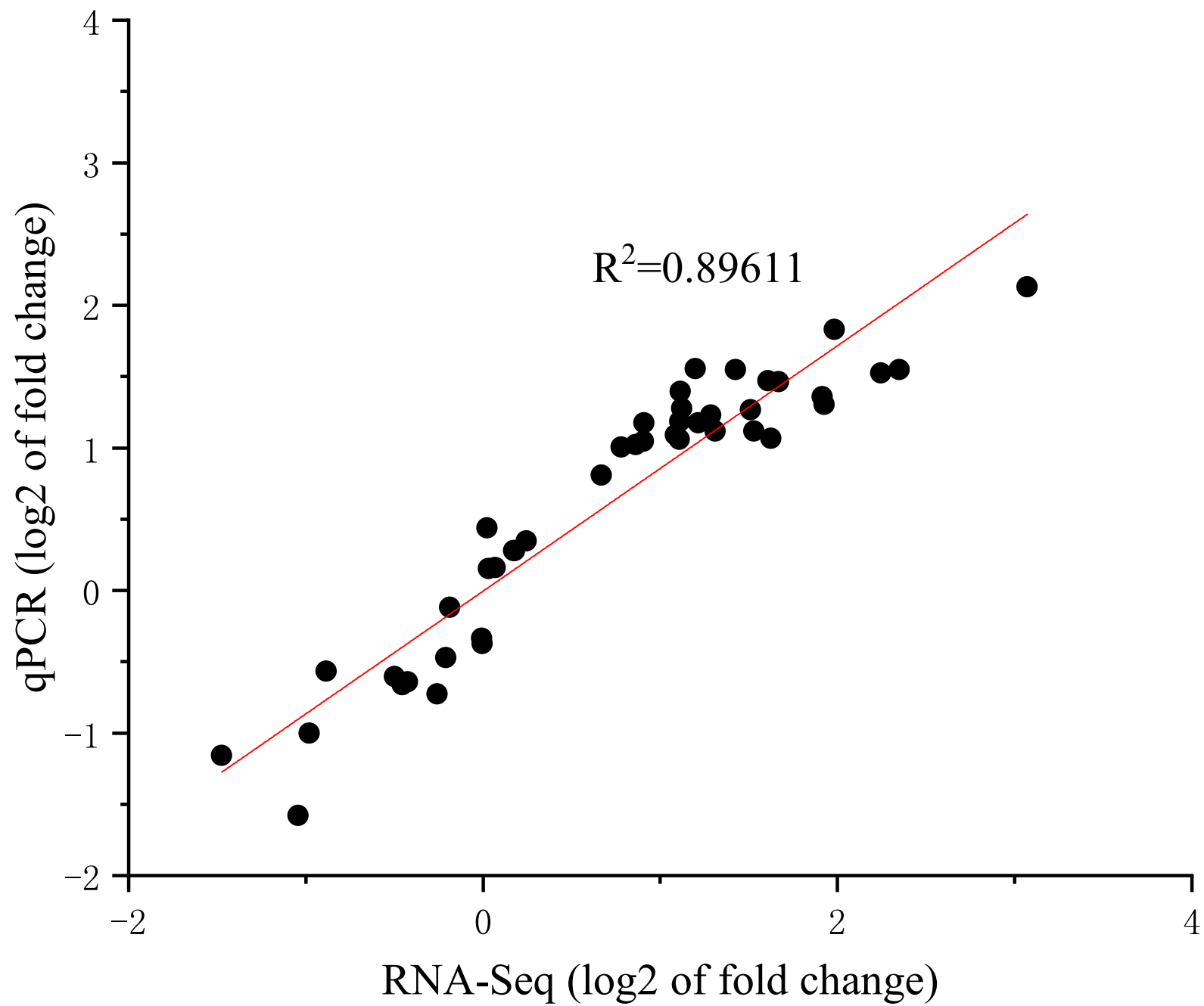

Supplement: S2 Fig — (PDF) [file pone.0231396.s002.pdf]
