# Supplementary material for: Identification and expression analysis of WRKY gene family under drought stress in peanut (Arachis hypogaea L.)
Source: PLoS One. 2020 Apr 9;15(4):e0231396. doi: 10.1371/journal.pone.0231396 (PMC7144997; doi:10.1371/journal.pone.0231396)

S1 File. Analysis and distribution of conserved motifs in peanut WRKY proteins.

Motif	Width	E value	Best possible match
1	29	1.2e-1036	[PA]R[VF][VSA][VF][QR][TA][RT]S[ED]VDILDDG[YC][RQ]WRKYGQKV[VA]K
2	30	7.0e-1006	[ES]D[IPL][PS][DES]DG[YW][NAS]WRKYGQK[PQ][IV]KGS[PE]YPR[SG]YY[KR]C
3	31	9.0e-998	GC[PN]V[RK]K[HRQ]V[EQ]R[ACL][SA]EDP[KSRT][IAM][VL][IV]TTYEGKHNH[PD][VL]P
4	26	4.2e-1007	DGY[NR]WRKYGQKQ[VI]KG[SN][PE][YF]PRSYY[RK]C[TS]
5	32	1.6e-940	[ST][SA][KPR]GCP[AV][RK]K[QH]V[EQ]R[SC]R[EDT]DP[TS][MI][LF][IV][VTI]TY[ET]GEHNH[PS]
6	29	9.8e-570	IL[DE]DGYQWRKYGQKV[VIT]K[GN][SN]P[YN]PR[SAN]YY[RK]C[ST]
7	32	2.0e-544	HPNCPVKKKVERS[HL]DG[QH]ITEI[IV]YKG[TQ]HNHPKP
8	27	7.9e-483	QGCP[AV][KT]K[QHR]V[QE]R[IS]Q[DE][DN]P[TP][LI][VY][IER]TTY[EY]GXH
9	11	3.8e-325	[GN][NS]P[NCH]PR[SA]YY[RK]C
10	42	7.00E-284	[LM]E[RE][VLM][KN][EA]EN[KQ][RK]L[RK]E[MT][LV][EN][QH][VI][CM][ESK][NS]YXALQM[QHK][LF]SN[LI][ML][QN][NK][KNQ]QXKEV[SE]

Numbers correspond to the motifs in Fig 3B

Motif 1

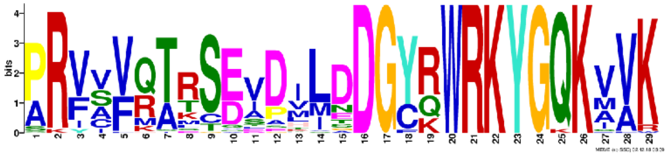

Motif 2

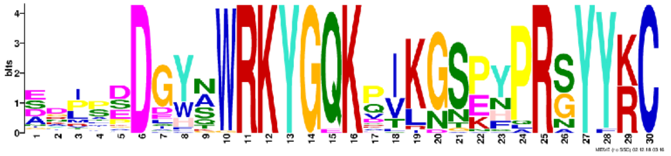

Motif 3

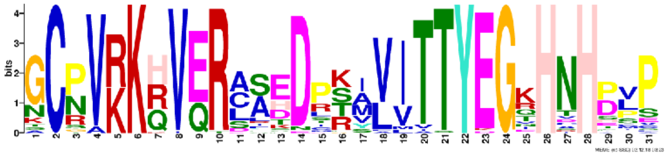

Motif 4

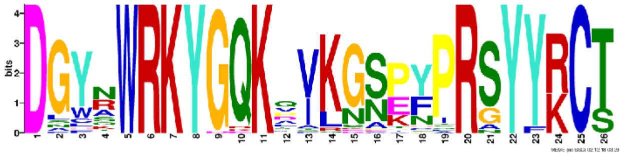

Motif 5

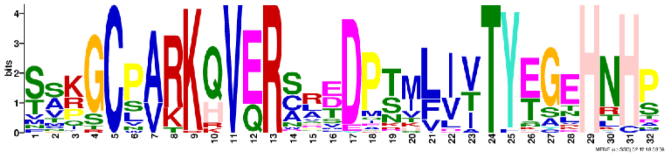

Motif 6

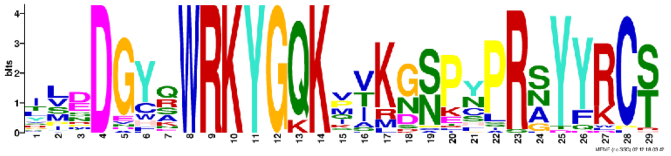

Motif 7

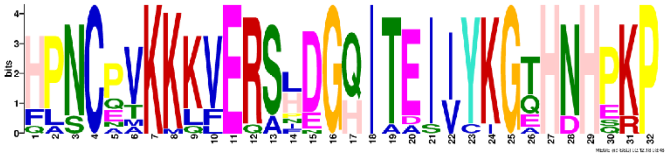

Motif 8

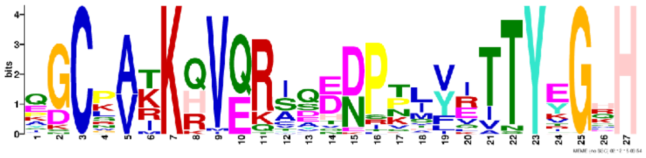

Motif 9

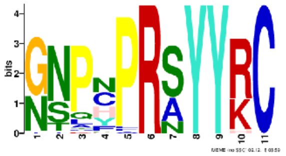

Motif 10

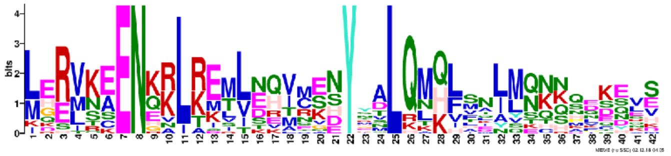

Supplement: S1 File — (PDF) [file pone.0231396.s003.pdf]
